# Surface Protected Organozirconium Catalyzes C─H Alumination of Saturated Hydrocarbons

**DOI:** 10.1002/anie.202511893

**Published:** 2025-07-30

**Authors:** Sayak Banerjee, Jessica Rodriguez, Marco Mais, Frédéric A. Perras, Aaron D. Sadow

**Affiliations:** ^1^ Department of Chemistry Iowa State University Ames IA 50011 United States; ^2^ Ames National Laboratory Iowa State University Ames IA 50011 United States

**Keywords:** Catalysis, CH activation, Hydrocarbon alumination, Methane functionalization, Surface organometallic chemistry

## Abstract

Surface grafted organozirconium catalyzes C─H/Et─Al exchange reactions, involving saturated hydrocarbons and AlEt_3_, to afford organoaluminum compounds and ethane. The Zr(O*^t^* Bu)_3_@SiO_2_‐Al_2_O_3–700_ (**1**) catalyst contains monopodal ≡SiO─Zr(O*^t^* Bu)_3_ and only a few residual silanols (<5%). Nonetheless, these silanols are the Achille's heel of **1**, providing a pathway for surface and catalyst degradation during catalysis, limiting the alkylaluminum yield and catalyst turnover. Support degradation, involving the cleavage of Si─O bonds by activated surface organometallics, is inhibited by capping silanols with ─SiMe_3_. Residual silanols in **1** react with allyltrimethylsilane, as determined by solid‐state ^13^C and ^29^Si nuclear magnetic resonance (NMR) spectroscopy, infrared (IR) spectroscopy, and reaction stoichiometry, to form Zr(O*^t^* Bu)_3_/SiMe_3_@SiO_2_‐Al_2_O_3–700_ (**2**), which is resistant to degradation by AlEt_3_. C─H alumination of dodecane catalyzed by **2** produces higher yields of the 1‐dodecylaluminum product in comparison to **1**, and in >95% selectivity. Additionally, methane undergoes **2**‐catalyzed C─H alumination, providing a route to AlMe_3_.

## Graphical abstract

C─H alumination of saturated aliphatic hydrocarbons is catalyzed by surface‐grafted organozirconium species, with high selectivity for the least‐hindered, terminal position, enabled by selective silylation of a small number of residual silanols in the catalyst. Exclusive mono‐alumination of methane is accomplished with more than 70 turnovers.

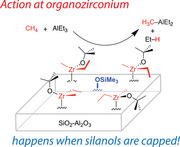

## Introduction

Alkylaluminums have an outsized role in large‐scale chemical manufacturing via transformations of unsaturated hydrocarbons, including as activators and chain transfer agents in olefin polymerization,^[^ ^1^ ^]^ as reactants in the oligomerization of ethylene via the Ziegler process,^[^ ^2^ ^]^ as well as in carboaluminations or hydroaluminations of alkenes.^[^ ^3^ ^]^ The organoaluminum products of these reactions are valuable due to their versatility and can be readily transformed into alcohols, carboxylic acids, or organohalides, or can be used in cross‐coupling, addition reactions, or olefin polymerization. In contrast to versatile, well‐developed C─H borylation chemistry,^[^ ^4^, ^5^, ^6^ ^]^ catalytic C─H alumination is limited to palladium‐catalyzed reactions of diketiminate‐supported aluminum(I) or dihydride reagents with arenes that give arylaluminum products.^[^ ^7^, ^8^ ^]^ The direct C─H alumination of saturated hydrocarbons could be valuable in commodity‐scale syntheses where inexpensive and abundant organoaluminum reactants would be advantageous. Triethylaluminum is a particularly appealing reactant for this type of process because it is prepared with perfect atom‐economy directly from ethylene, hydrogen, and aluminum, the most abundant metal in the earth's crust.^[^ ^9^ ^]^ The by‐product of C─H alumination using AlEt_3_ is ethane, which can be cracked into ethylene and hydrogen, and could be recovered and reused to make more AlEt_3_ in an atom‐efficient, cyclical process. Unlike exothermic oxidative C─H functionalization processes where oxidized products are often more reactive than alkanes themselves,^[^ ^10^ ^]^ alkyl group exchange at aluminum is nearly thermoneutral, and products are less likely to undergo secondary functionalization steps.

Our choice of zirconium as the active metal site for a first‐generation C─H alumination catalyst was based upon its pre‐eminent role in existing aluminum–carbon bond forming processes, such as carboalumination, and the high reactivity of electrophilic early organo‐transition metal compounds in C─H bond activation of aliphatic and aromatic hydrocarbons via σ‐bond metathesis.^[^ ^11^, ^12^, ^13^, ^14^ ^]^ C─H bond cleavage via σ‐bond metathesis has been proposed as the key step in a few processes catalyzed by *d* ^0^ metal centers, including hydrocarbon hydrogenolysis,^[^ ^15^, ^16^, ^17^, ^18^ ^]^ propane homologation,^[^ ^19^ ^]^ dehydrogenative silylation,^[^ ^20^, ^21^, ^22^ ^]^ hydroarylation,^[^ ^23^, ^24^, ^25^ ^]^ and dehydrogenative borylation.^[^ ^26^, ^27^, ^28^, ^29^, ^30^ ^]^ In addition, air‐stable zirconium precatalysts are activated for olefin polymerization by alkylaluminum compounds,^[^ ^31^ ^]^ and we had found that an air‐exposed, deactivated neopentoxyzirconium species could be re‐activated for catalytic deconstruction of polyolefins in the presence of Al*^i^* Bu_3_^[^ ^32^ ^]^ These ideas led us to the first example of this C─H/Et─Al exchange to achieve the C─H alumination of aliphatic hydrocarbons, (Equation 1)^[^ ^33^ ^]^ which used an air‐stable, surface‐supported alkoxyzirconium complex Zr(O*^t^* Bu)_3_@SiO_2_‐Al_2_O_3–700_ (**1**) as a precursor for in situ generation of the active organometallic catalyst.

(1)

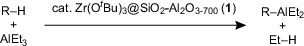

The surface organometallic chemistry (SOMC) catalyst likely benefits from the high reactivity of surface‐supported organozirconium species in C─H activations of hydrocarbons, including methane.^[^ ^34^, ^35^ ^]^ SOMC catalysts on silica–alumina have been reported to be even more active than their silica‐supported counterparts.^[^ ^18^, ^19^, ^36^ ^]^ Silica–alumina was also superior as a support compared to silica in zirconium‐catalyzed hydrocarbon alumination, possibly because it appeared to be less susceptible to AlEt_3_‐mediated degradation.^[^ ^33^ ^]^ Even so, C─H alumination reactions using silica‐ and silica–alumina‐supported systems led to the extrusion of soluble ethylsilyl species and the partial dissolution of the support material. These side reactions undoubtedly involve ethyl species attacking silicon–oxygen bonds. We sought to identify the mechanisms of this attack that lead to the surface degradation and to prevent this side reaction. Not only would that immediately improve the selectivity of C─H alumination catalysis, insight from these experiments would also help establish a relationship between chemistry occurring on the support and the activity of the catalytic site.

Our approach is inspired by the silylation of metal oxides to allow catalysis in aqueous media, where dissolution of the support can be an issue. For example, reaction of zeolites with trialkylchlorosilanes prevents hydrolytic deactivation in hydrothermal catalytic reactions.^[^ ^37^ ^]^ Likewise, the partial silylation of hydroxy groups of silica‐supported titanium or tantalum catalysts improves yields of epoxidations using H_2_O_2_ in water.^[^ ^38^, ^39^ ^]^ In addition to limiting silica hydrolysis in the latter case, increased catalytic activity is attributed to the formation of M─OSiMe_3_ moieties. We sought to avoid such reactions at the zirconium pre‐catalyst center, since the alkoxide group affected catalytic activity.^[^ ^33^ ^]^ In addition, the oxophilicity of aluminum, its nucleophilic alkyl groups, and the non‐polar environment of C─H alumination catalysis are expected to create distinct challenges compared to the established aqueous chemistry. In this vein, the high reactivity of strong Brønsted acid sites with Si_2_Me_6_ has been leveraged to selectively silylate sulfated zirconia in the presence of grafted ruthenium hydrosilylation catalysts.^[^ ^40^ ^]^ Capping only the Brønsted acidic sites in SiO_2_‐Al_2_O_3_ or sulfated zirconia,^[^ ^41^ ^]^ however, is not guaranteed to eliminate the side reactions involving alkylaluminums that leads to degradation of **1**. Also, silylation of surface silanols with Me_3_SiBr, prior to introduction of Cl_2_(PCy_3_)_2_Ru(═CHPh), allowed coordination of Grubbs catalyst to silica‐immobilized *N*‐heterocyclic carbenes.^[^ ^42^, ^43^ ^]^ Because we found that chloride or amide zirconium precatalysts were less active for C─H alumination than tert‐butoxyzirconium, halide‐ and amine‐free silylating agents were pursued. Here, we employ H_2_C═CHCH_2_SiMe_3_^[^ ^44^ ^]^ as a reagent for the selective silylation of residual surface hydroxy groups in **1**, to minimize the degradation of the catalyst support. That has, in turn, enabled systematic studies that led to increased catalytic turnovers, higher yields, and near‐perfect selectivity for alkylaluminum products.

## Results and Discussion

### AlEt_3_ Deactivates Zr(O*^t^* Bu)_3_@SiO_2_‐Al_2_O_3–700_

We briefly describe the unprotected supported organozirconium material's composition, structure, and C−H alumination catalysis, with respect to residual silanols and for comparison to the silylated analogues. The reaction of Zr(O*^t^* Bu)_4_ in pentane and SiO_2_‐Al_2_O_3_ partially dehydroxylated at 700 °C (0.66 ± 0.02 mmol OH·g^−1^) affords **1** and *^t^* BuOH, as previously reported (Equation 2).^[^ ^33^ ^]^

(2)

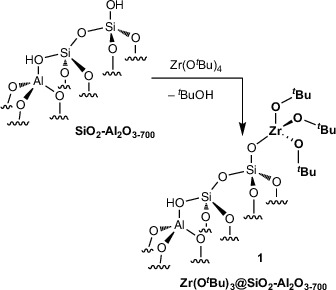

**Figure 1:**
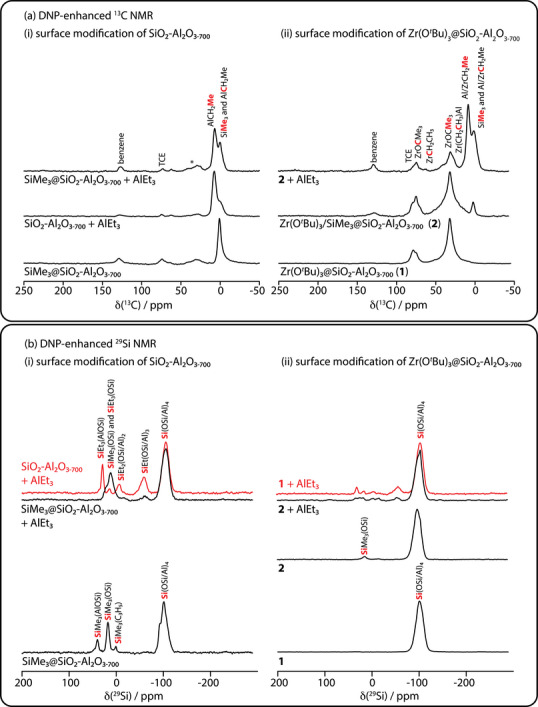
DNP‐enhanced ^13^C (a) and ^29^Si (b) CPMAS NMR spectra measured for SiO_2_‐Al_2_O_3–700_ (i) and Zr(O*^t^* Bu)_3_@SiO_2_‐Al_2_O_3–700_ (ii) materials treated with 60 equiv of AlEt_3_ and/or allyltrimethylsilane, as indicated. In (a) an asterisk denotes the presence of aliphatic impurities. SiO_2_‐Al_2_O_3–700_ and AlEt_3_ or SiMe_3_@SiO_2_‐Al_2_O_3–700_ and AlEt_3_ were heated at 150 °C in dodecane prior to analysis.

The 0.59 ± 0.05 mmol *^t^* BuOH·g^−1^ formed in this grafting reaction and the 0.63 ± 0.03 mmol Zr·g^−1^ in **1**, measured using inductively coupled plasma‐optical emission spectroscopy (ICP‐OES), nearly matched the accessible OH loading of the initial SiO_2_‐Al_2_O_3–700_ material and supported the formulation of surface species as ≡SiO─Zr(O*^t^* Bu)_3_. Quantification of the basic ligands in **1** by formic acid protonolysis as ∼1.77 mmol O*^t^* Bu·g^−1^ further indicated, along with the Zr loading, that there were 2.9 ± 0.2 O*^t^* Bu groups per Zr center. The DNP‐enhanced ^13^C CPMAS NMR spectrum of **1** (Figure 1) contained signals at 75 and 33 ppm assigned to ZrO*C*Me_3_ and ZrOC*Me*_3_, respectively.^[^ ^45^ ^]^ The *ν*_CH_ bands in the infrared spectrum of **1**, measured in DRIFTS configuration, provided evidence for the presence of CMe_3_ groups. The typical sharp band at 3748 cm^−1^ assigned to isolated silanols (*ν*_SiO–H_) in the spectrum of partially dehydroxylated SiO_2_‐Al_2_O_3‐700_, however, was not detected in the IR spectrum of **1**. Instead, a broad signal at 3600 cm^−1^ indicates that surface OH species hydrogen bond, with tert‐butoxyzirconium species or a restructured SiO_2_‐Al_2_O_3_ surface.

We previously reported that **1** is active for the catalytic C─H alumination of *n‐*dodecane.^[^ ^33^ ^]^ For example, heating **1** (0.019 mmol Zr), AlEt_3_ (0.57 mmol), and *n*‐dodecane (1.32 mmol) at 150 °C gives 15 turnovers to *n*‐dodecan‐1‐ol (0.289 mmol, 51% with respect to limiting AlEt_3_, 22% yield with respect to *n*‐dodecane) after workup. Reaction workup by quenching with O_2_ at 0 °C followed by hydrolysis under basic conditions affords *n*‐dodecan‐1‐ol as the only product, while acidic conditions give isomerization to secondary alcohols.

**Figure 2:**
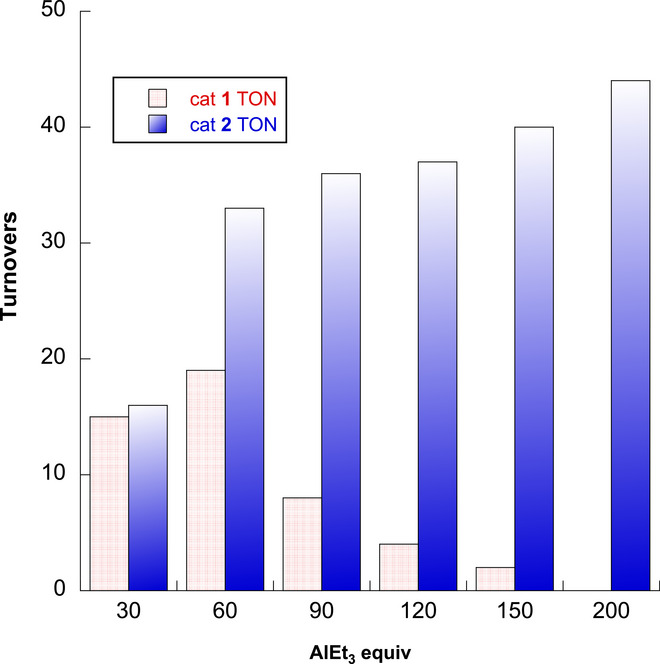
Decreasing or increasing turnovers for dodecylaluminum formation with increasing AlEt_3_ equivalents using catalysts **1** (red patterned bars) or **2** (blue filled bars), respectively.

Because AlEt_3_ is the limiting reagent under these conditions, more AlEt_3_ could lead to higher turnovers and higher yield with respect to dodecane (Figure 2). That idea is only partly supported by the experiments. As the AlEt_3_ is increased from 0.57 mmol (30 equiv with respect to Zr) to 1.14 mmol (60 equiv with respect to Zr), the yield of dodecan‐1‐ol increases to 0.367 mmol (32% with respect to limiting AlEt_3_, 28% with respect to n‐dodecane, 19 turnovers). Additional AlEt_3_ in these experiments, unfortunately, results in poorer yields and fewer turnovers, with 150 equiv providing only two turnovers. For catalyst **1**, 60 equiv of AlEt_3_ gives the highest yield of dodecan‐1‐ol. At the same time, the formation of soluble ethylsilyl species (∼0.41 mmol ethyl·g^−1^ from 60 equiv of AlEt_3_, determined from integrated ^1^H NMR signals relative to a standard of known concentration) is increased with higher concentrations of AlEt_3_. Thus, the effects of AlEt_3_ on dodecylaluminum and ethylsilane yields follow opposite trends with **1** as the catalyst, suggesting that support degradation could be limiting the catalytic conversion.

After catalysis, the solid material turned dark brown, and the Al loading increased to 0.48 mmol·g^−1^ from 0.44 mmol g^−1^ in **1**. Zr loading (0.61 mmol·g^−1^) in the dark brown material matched that in **1**. In addition, ethylsilyl groups (≡SiEt, ═SiEt_2_, ─SiEt_3_) were observed on the surface of SiO_2_‐Al_2_O_3_ by DNP‐enhanced ^29^Si and ^13^C CPMAS NMR spectroscopy (Figure 1). We did not detect dodecan‐1‐ol after workup using the dark‐brown material as a recycled catalyst, in experiments performed by decanting the liquids from a first experiment and adding fresh *n*‐dodecane and AlEt_3_ (60 equiv with respect to Zr) to the brown solid material. Rather than giving higher yields of dodecylaluminum, adding more AlEt_3_ appears to accelerate ethylsilyl formation.

The effects of AlEt_3_ on catalytic activity could be related to its reactions at the zirconium sites or at residual silanols on the support. **1^80%^** was synthesized, with Zr(O*^t^* Bu)_4_ grafted at only ∼80% of the OHs, to investigate how decreased Zr or increased OH loadings affect the catalysis. The Zr loading in **1^80%^** was 0.46 ± 0.02 mmol·g^−1^ by ICP‐OES, corresponding to 80% of the original protic sites in SiO_2_‐Al_2_O_3–700_ and 75% of the Zr loading of **1**. Excess MgBn_2_ was protonated by the residual protic sites in **1^80%^** to generate toluene, which was quantified to determine the hydroxy loading of 0.114 mmol OH·g^−1^ (i.e., ∼20% of protic sites in the original SiO_2_‐Al_2_O_3–700_). The DRIFTS of **1^80%^** contained a broad signal at 3598 cm^−1^ assigned to hydrogen‐bonded silanols or bridging ≡Si(≡Al)OH,^[^ ^46^ ^]^ while the peak assigned to isolated silanols was not observed. Under comparable conditions using equivalent mol % zirconium, *n*‐dodecane (1.32 mmol), and AlEt_3_ (30 or 60 equiv), precatalyst **1^80%^** was much less active than **1** and yielded only 11% or 2% dodecan‐1‐ol (8 or 1.4 turnovers), respectively. In addition, much more ethylsilyl species (2.7 mmol EtSi·g^−1^) were detected in the ^1^H NMR spectrum of the deep brown reaction mixture using **1^80%^** than in reactions catalyzed by **1**. Thus, the presence of accessible protic sites in **1^80%^** leads to greater catalyst deactivation by AlEt_3_ than **1** itself.

### Reaction of AlEt_3_ with SiO_2_‐Al_2_O_3_ and Surface‐Protected SiO_2_‐Al_2_O_3_

Because silanols appeared to be involved in the deactivation of **1** under catalytic conditions, we investigated the degradation of SiO_2_‐Al_2_O_3–700_ itself by AlEt_3_, in the absence of grafted zirconium species, to develop strategies to improve catalytic turnovers and product yields. The reaction of excess AlEt_3_ (30 equiv) and partially dehydroxylated SiO_2_‐Al_2_O_3–700_ (20 mg; 0.66 ± 0.02 mmol OH·g^−1^ measured by reaction with MgBn_2_) at room temperature generates 0.66 ± 0.02 mmol ethane·g^−1^ (GC‐FID). The ca. 0.66 mmol AlEt_3_·g^−1^ consumed in this reaction, estimated by integration of residual ethyl signals in the ^1^H NMR spectrum of the reaction mixture, was consistent with quantitative reaction of the ─OH groups. Although the structures of the supported Al species were not determined in this work, it is reasonable to assume that they adopt similar configurations as seen on silica (Equation 3). For instance, (≡SiO)_2_–Al_2_Et_4_ sites are formed on SiO_2_ dehydroxylated under vacuum at 500 °C.^[^ ^47^ ^]^

(3)

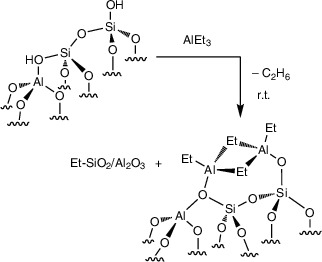

Ethylsilyl species were not detected in solution by ^1^H NMR spectroscopy from these room temperature reaction conditions. Heating a mixture of SiO_2_‐Al_2_O_3–700_ (40 mg) and AlEt_3_ (1.26 mmol) in *n*‐dodecane at 150 °C for 12 h (Equation 4; conditions typically used for catalytic C─H alumination) forms, in addition to ethane, soluble species with ethylsilyl groups (1.20 mmol·g^−1^) corresponding to ∼2× the initial silanols present in SiO_2_‐Al_2_O_3–700_ and equivalently 2× the amount of AlEt_3_ that was grafted onto the surface at room temperature. ^29^Si dynamic nuclear polarization (DNP)‐enhanced cross polarization magic‐angle spinning (CPMAS) NMR of SiO_2_‐Al_2_O_3–700_ heated with AlEt_3_ at 150 °C shows that Et_3_Si(OSi/Al) (23 and 8 ppm), Et_2_Si(OSi/Al)_2_ (−13 ppm), and EtSi(OSi/Al)_3_ (−66 ppm) surface sites formed (see Figure 1). Similar ^29^Si NMR signals were observed in SiO_2_‐Al_2_O_3_ samples that were allowed to react with AlEt_3_ at room temperature. Thus, silicon has been alkylated by its reaction with AlEt_3_.

(4)

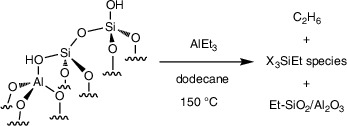

The degradation of silica–alumina under these conditions could involve either the direct alkylation of surface siloxanes by AlEt_3_ from solution, covalently grafted ethylaluminum species attacking nearby surface siloxanes, or grafted ethylaluminum sites mediating the reaction of solution‐phase AlEt_3_ and siloxanes. The first possible pathway was tested by heating AlEt_3_ with a trimethylsilyl‐capped silica–alumina, which had a very low loading of surface silanols to limit the sites for AlEt_3_ grafting. The trimethylsilyl‐protected silica–alumina Me_3_Si@SiO_2_‐Al_2_O_3–700_ itself was prepared by treatment of SiO_2_‐Al_2_O_3–700_ with H_2_C═CHCH_2_SiMe_3_ at 150 °C in pentane in a sealed pressure vessel (Equation 5).^[^ ^44^ ^]^ This capping reaction evolved 0.65 ± 0.03 mmol propylene·g^−1^, corresponding to quantitative consumption of the accessible surface silanols. That assessment was also supported by reaction of Me_3_Si@SiO_2_‐Al_2_O_3–700_ with MgBn_2_, which revealed that only ∼0.004 mmol OH·g^−1^ remained in the material.

(5)

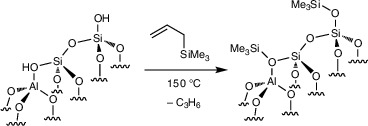

DNP‐enhanced ^29^Si CPMAS NMR measurements of Me_3_Si@SiO_2_‐Al_2_O_3–700_ (Figure 1) revealed surface ≡SiO─SiMe_3_ (18 ppm) and bridging ≡Si(≡Al)O─SiMe_3_ (41 ppm) sites, with the latter being shifted due to the more polar bonding to the support.^[^ ^48^ ^]^ Semi‐quantitative integration of these sites suggests that ca. 1/3 of the surface hydroxys are of the bridging variety. This ratio agrees reasonably well with the 27% Al loading of the SiO_2_‐Al_2_O_3_. The surface SiMe_3_ groups are also evident by DNP‐enhanced ^13^C CPMAS NMR at 2 ppm.

The reaction of Me_3_Si@SiO_2_‐Al_2_O_3–700_ with excess AlEt_3_ (30 equiv) at room temperature evolved only ∼0.002 mmol ethane·g^−1^. The ^13^C and ^29^Si SSNMR spectra of the AlEt_3_‐treated Me_3_Si@SiO_2_‐Al_2_O_3_ were largely identical to Me_3_Si@SiO_2_‐Al_2_O_3_ itself, displaying surface SiMe_3_ in addition to a small amount of new AlEt sites. Notably, surface alkylation products were reduced by approximately one order of magnitude, as compared to that seen in unprotected SiO_2_‐Al_2_O_3–700_ (Figure 1). In further contrast to the degradation of SiO_2_‐Al_2_O_3–700_ in the presence of AlEt_3_, soluble ethylsilyl species were not detected after reaction of excess AlEt_3_ and Me_3_Si@SiO_2_‐Al_2_O_3–700_ in *n*‐dodecane at 150 °C for 12 h. Thus, ≡Si─O─Si≡ and ≡Si─O─SiMe_3_ surface linkages are inert to direct alkylation by AlEt_3_ from solution.

The second possible pathway for support degradation, in which covalently grafted ethylaluminum surface moieties extrude ethylsilyl species, was ruled out by isolating AlEt_3_‐treated SiO_2_‐Al_2_O_3–700_ and then heating that material in dodecane at 150 °C for 12 h. Under these conditions in the absence of free AlEt_3_, soluble ethylsilyl species were not detected by GC or NMR spectroscopy. Heating the isolated AlEt_3_‐treated SiO_2_‐Al_2_O_3–700_ with free AlEt_3_ in dodecane, however, produced soluble ethylsilyl species. Thus, surface degradation, at least in the absence of other surface organometallics, involves reaction of solution‐phase AlEt_3_ with surface‐grafted organoaluminum sites. These results further show that support degradation from reaction with AlEt_3_ does not require zirconium as a catalyst or mediator, and that capping of protic sites passivates the surface against reaction with AlEt_3_ by blocking the grafting step. These conclusions suggest that protecting any residual ≡SiOH and ≡Si(≡Al)OH grafting sites in **1** might inhibit support degradation and increase turnover numbers in the catalytic C─H alumination.

### Silanol Capping to Improve Catalytic C─H Alumination

Because grafting of AlEt_3_ onto SiO_2_‐Al_2_O_3–700_ is part of a pathway for surface degradation, and experiments on zirconium‐free SiO_2_‐Al_2_O_3–700_ show its reactions with AlEt_3_ can be blocked by surface silylation, the capping of residual protic sites in **1** or **1^80%^** could be a strategy to improve C─H alumination catalysis. Me_3_SiCl, as a typical silylating agent, slowly reacts with **1** at room temperature in benzene‐*d*_6_ to form *^t^* BuOH and *^t^* BuOSiMe_3_ along with (presumably) chlorozirconium surface species. Note that Zr(O*^t^* Bu)_4_ itself reacts with Me_3_SiCl slowly in refluxing benzene‐*d*_6_ or toluene‐*d*_8_ (∼8% Zr(O*^t^* Bu)_4_ conversion after 3 h). In contrast, H_2_C═CHCH_2_SiMe_3_ and Zr(O*^t^* Bu)_4_ return only starting materials under that condition. Thus, H_2_C═CHCH_2_SiMe_3_ was selected as the silylating agent to avoid side reactions of the ≡SiO─Zr(O*^t^* Bu)_3_ pre‐catalyst site.

**Scheme 1:**
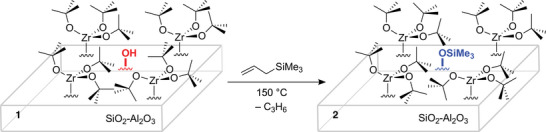
Selective silylation of residual surface OH in **1** in the presence of grafted tert‐butoxyzirconium species to form **2**.

**Figure 3:**
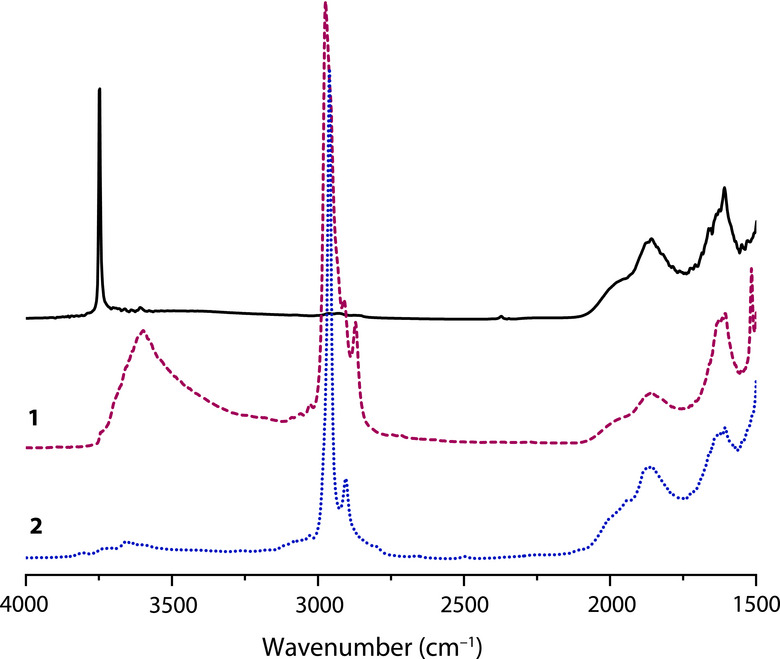
DRIFTS of SiO_2_‐Al_2_O_3–700_ (black continuous), **1** (grey dashed) and **2** (blue dotted), normalized to the silicon framework signal at 1610 cm^−1^.

The reaction of **1** (0.63 mmol Zr·g^−1^) and H_2_C═CHCH_2_SiMe_3_ (0.164 mmol) at 150 °C gave **Zr(O*^t^* Bu)_3_/SiMe_3_@SiO_2_‐Al_2_O_3–700_** (**2**; Scheme 1) and generated propylene (0.07 ± 0.03 mmol·g^−1^, quantified by GC), which matched the estimated loading of residual accessible silanols in **1** (0.04–0.07 mmol·g^−1^). **1^80%^** was similarly treated with H_2_C═CHCH_2_SiMe_3_ to make **2^80%^**. DNP‐enhanced ^13^C and ^29^Si CPMAS NMR spectra of **2** detected O─SiMe_3_ as minor surface species, in agreement with the low residual concentration of silanols in **1** (Figure 1). Notably, the DRIFTS of **2** did not contain the broad, weak feature at 3600 cm^−1^ associated with the bridging silanols in **1** (Figure 3). The comparison of **1^80%^** and **2^80%^** using DRIFTS also showed the consumption of residual OHs (Figures S4 and S5).

In these capping experiments, we did not detect *^t^* BuOSiMe_3_, which could have formed from an unwanted reaction of ZrO*^t^* Bu moieties and H_2_C═CHCH_2_SiMe_3_. The Zr loading in **2** of 0.59 ± 0.03 mmol·g^−1^ was equivalent to the loading of Zr in **1**. In addition, reaction of **2** with formic acid released 1.65 mmol of *^t^* BuOH·g^−1^, corresponding to 2.8 O*^t^* Bu per Zr, within error of expectation. The Zr loading in the **2^80%^** material was 0.44 ± 0.02 mmol·g^−1^. Thus, the pre‐catalyst sites are equivalent in **1** and **2**.

The reaction of **1** and AlEt_3_ (5 equiv with respect to Zr) in benzene‐*d*_6_ at room temperature over 4 h forms *^t^* BuOAlEt_2_, ethane (0.058 mmol·g^−1^) and a deep brown solid. 18% of the total tert‐butoxy (0.054 mmol·g^−1^) in **1** transfer to soluble aluminum species, suggesting that the material contains ≡SiO─ZrEt(O*^t^* Bu)_2_ and ≡SiO─Zr(O*^t^* Bu)_3_ surface species in a ca. 1:1 ratio under these conditions (Equation 6).

(6)

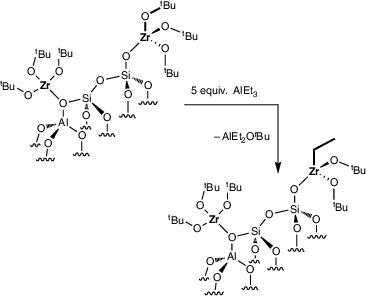

While transfer of tert‐butoxy groups is barely detectable with only 2 equiv of AlEt_3_, the extent of exchange of tert‐butoxy and ethyl groups increases with the addition of more AlEt_3_. Reaction of 60 equiv of AlEt_3_ and **1** gives 2 equiv of *^t^* BuOAlEt_2_ relative to Zr, which implies that doubly‐exchanged surface species, such as ≡SiO─ZrEt_2_O*^t^* Bu, are formed (see below). Although the analysis cannot rule out mixtures also containing ≡SiO─ZrEt(O^*t*^Bu)_2_ and ≡SiO─ZrEt_3_ in a 1:1 ratio, the (formally) dialkylated zirconium species is more likely because of the large excess of AlEt_3_. Ethane is formed as a product of the protonation of AlEt_3_ with the residual ─OH groups in **1**, as in the reaction of AlEt_3_ and SiO_2_‐Al_2_O_3–700_ above. The ethane yield (0.06 or 0.08 mmol·g^−1^) from reaction of **1** with 5 or 60 equiv of AlEt_3_, respectively, equaled the estimated silanols in **1** (0.07 mmol·g^−1^). ICP‐OES of **1** treated with 5 or 60 equiv of AlEt_3_ at room temperature revealed increased Al loading (0.37 ± 0.04 or 0.39 ± 0.03 mmol·g^−1^, respectively) compared to SiO_2_‐Al_2_O_3–700_ (0.31 ± 0.02 mmol·g^−1^) and **1** (0.29 ± 0.04 mmol·g^−1^). The Zr loading of 0.58 ± 0.03 or 0.57 ± 0.03 mmol·g^−1^ after treatment with 5 or 60 equiv of AlEt_3_ was comparable to **1** itself, suggesting that Zr was not leached from the surface by the alkylaluminum.

Reactions of **2** with 5–200 equivalents of AlEt_3_ with respect to Zr also form *tert*‐butoxyethylaluminum species in similar yields as in reactions of **1** above, implying similar ethylated zirconium species. The comparison of **1** and **2** in their reactions with 5 equiv of AlEt_3_ showed similar *^t^* BuOAlEt_2_ formation, yet **2** turned light yellow and produced only 0.008 mmol EtH·g^−1^, approximately one order of magnitude less than from the corresponding reaction of **1** (0.058 mmol·g^−1^). Increases in AlEt_3_ (i.e., at 60, 150, or 200 equiv of AlEt_3_ with respect to Zr) also transferred ∼66% of the tert‐butoxy groups from **2** to Al, produced 0.034 mmol EtH·g^−1^, and afforded a yellow material. The surface species ≡SiO─ZrEt_2_O*^t^* Bu in this material is suggested by the reaction stoichiometry, however, these are likely alkylaluminum adducts such as ≡SiO─ZrEt(Et_2_AlEt_2_)O*^t^* Bu (Equation 7) as a result of the large excess of AlEt_3_. In addition, [Zr]Et_2_ could react by β‐alkyl abstraction. These tentative assignments are supported by ^13^C SSNMR signals at 55 ppm (Zr**C**H_2_CH_3_) and a shoulder at 20 ppm (ZrCH_2_ **C**H_3_ and ZrCH_2_(**C**H_3_)Al), while the signal from Zr**C**H_2_(CH_3_)Al would overlap with AlCH_2_CH_3_ at 9 ppm (Figure 1), based on comparisons to Cp_2_HfEt_2_ and [Cp_2_HfEt_2_AlEt_2_][B(C_6_F_5_)_4_].^[^ ^49^ ^]^

(7)

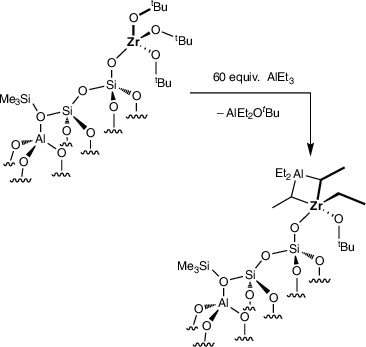

ICP‐OES of **2** treated with 60 equiv of AlEt_3_ at room temperature revealed comparable Al loading (0.31 ± 0.04 mol·g^−1^, respectively) as SiO_2_‐Al_2_O_3–700_ (0.31 ± 0.02 mmol·g^−1^) and **2** (0.29 ± 0.03 mmol·g^−1^). The Zr loading of 0.57 ± 0.04 mmol·g^−1^ after treatment with 60 equiv of AlEt_3_ was comparable to **2** itself, further suggesting that Zr was not leached from the surface by the alkylaluminum treatments of **2** (as in **1**). Comparison of **1** and **2** treated with 60 equiv of AlEt_3_ using DNP‐enhanced ^29^Si CPMAS NMR revealed less surface alkylation in the reaction of the latter material, and significantly less than seen with the base support (Figure 1). Based on the above results, **2** was studied as a catalyst for dodecane alumination.

### Catalytic C─H Alumination of *n*‐Dodecane by 2

The yield and turnovers for dodecan‐1‐ol formation increased with increasing AlEt_3_ (Figure 2, blue bars), in contrast to the deactivation of **1** at high AlEt_3_. In the reaction employing 30 equiv of AlEt_3_ (with respect to Zr), catalyst **2** afforded a slightly higher yield of dodecan‐1‐ol (0.308 mmol, 16 turnovers) than catalyst **1** (0.289 mmol, 15 turnovers). Doubling AlEt_3_ to 60 equiv with catalyst **2** produced 0.621 mmol of dodecan‐1‐ol, essentially doubling the turnovers to 33 and yield to 47% (with respect to dodecane). With catalyst **1**, doubling AlEt_3_ from 30 to 60 equiv increased the turnovers from 15 to only 19. Experiments decreasing the loading of **2** with respect to dodecane, rather than increasing the equivalents of AlEt_3_, gave lower yields. Thus, reactions using 0.5 mol% Zr, compared to the 1.4 mol% catalyst in the above experiments, provided only 10.1% yield of dodecan‐1‐ol (corresponding to 19 turnovers). Moreover, **2^80%^** gave comparable results as **2** under similar conditions. For instance, 0.019 mmol of Zr (in material **2^80%^**) and 60 equiv of AlEt_3_ (with respect to Zr), gave 48% (0.636 mmol) conversion of dodecane and 46% yield (0.611 mmol) of dodecan‐1‐ol, corresponding to 32 turnovers and 96.1% selectivity. Still, lower mol % Zr with **2**, or lower loading of Zr in **2^80%^**, followed the trend of increasing turnovers with increasing equivalents of AlEt_3_ established for 1.4 mol% **2**. In contrast, uncapped **1^80%^** was a poor C─H alumination catalyst under these conditions, and experiments using 0.5 mol% Zr of catalyst **1** did not provide detectable dodecan‐1‐ol.

The turnovers and dodecan‐1‐ol yield in reactions catalyzed by **2** increase monotonically with increasing AlEt_3_ in the reactor, up to a TON of 44 and 63% yield with 200 equiv of AlEt_3_ (Equation 8). Even at this higher dodecane conversion, selectivity for the product is 97%.

(8)

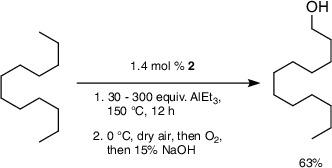

Only traces of ethylsilyl species (0.008 mmol) were observed by ^1^H NMR spectroscopy when 200 equiv of AlEt_3_ were used, which is two orders of magnitude less than observed with **1** as catalyst. Experiments employing 300 equiv of AlEt_3_ with respect to Zr afforded equivalent yields of dodecan‐1‐ol (0.839 mmol with respect to n‐dodecane, TON of 44) as experiments using 200 equivalents. The yield does not increase with longer reaction times. The higher yields and turnovers with **2** provide a stark contrast to the precipitous fall of dodecan‐1‐ol yield and turnovers with increasing AlEt_3_ using catalyst **1**. That is, the large excess of AlEt_3_ does not lead to deactivation of the active catalyst from **2** (or **2^80%^**) because yields do not decrease as they do with **1**. Thus, we conclude that Me_3_SiCH_2_CH═CH_2_ treatment increases the lifetime of **2** compared to **1**. Nonetheless, the yield of dodecan‐1‐ol reaches a plateau of ∼65% at the higher concentrations of AlEt_3_.

The color of the material from **2** was pale yellow after the catalytic reaction, in contrast to the change in color of **1** to brown. Addition of a fresh portion of dodecane (1.32 mmol) and fresh AlEt_3_ (60 equiv with respect to Zr) to the used sample of **2** yielded a small amount of dodecan‐1‐ol after workup (0.106 mmol, 8% yield with respect to n‐dodecane, ∼6 turnovers). Formation of ethyl silyl species (0.026 mmol) from recycled **2** greatly increased compared to that from the fresh catalyst. Although there was a clear degradation of the catalytic material after the first alumination experiment, addition of more **2** to the reaction mixture containing excess AlEt_3_ or transfer of the reaction solution to a fresh portion of **2** (preactivated with AlEt_3_) also did not lead to higher yield of dodecan‐1‐ol (after workup). Because high AlEt_3_ did not appear to inhibit catalysis, this result suggested that dodecyldiethylaluminum was inhibiting the C─H alumination.

Yields should improve under diluted conditions if the product is competing for coordination to the active sites. Initial attempts using toluene or toluene‐*d*_8_ as a solvent did not provide the dodecan‐1‐ol product (or benzyl alcohol or phenol products), and dodecane was recovered from the experiment. Because **1** was previously observed to react with a large kinetic isotope effect,^[^ ^33^ ^]^ such that dodecane‐*d*_26_ did not react under alumination conditions, the deuterated aliphatic hydrocarbon was used as the solvent to dilute the dodecane. As with **1**, **2** did not provide detectable alumination of dodecane‐*d*_26_, and a 1:1 mixture of dodecane and dodecane‐*d*_26_ gave only linear 1‐C_12_H_25_OH. Neither dodecan‐1‐ol‐*d*_25_ nor any isotopologues of dodecane or dodecan‐1‐ol (C_12_H_x_D_26−x_ or C_12_H_x_D_25−x_OH) were detected by GC‐MS or ^2^H NMR spectroscopy after workup, ruling out any H/D exchange, thereby suggesting that C─D bonds are not cleaved in the catalytic transformation. These observations indicate that a very large isotope effect governs the activation of C─H bonds in this system.

We then applied dodecane‐*d*_26_ as a diluent to study catalytic C─H alumination of dodecane. A 1:1 mixture of dodecane (0.66 mmol) and dodecane‐*d*_26_ (0.66 mmol) was subjected to 120 equiv AlEt_3_ in the presence of **2** at 150 °C for 12 h. The catalysis resulted in the conversion of C_12_H_26_ to linear dodecan‐1‐ol (72% yield with respect to C_12_H_26_, 25 turnovers) as the primary product. The turnovers decreased compared to standard conditions, limited in part by the low initial concentration of dodecane. Therefore, a companion dilution experiment was conducted with the amount of dodecane matching standard conditions (1.32 mmol) and doubled total volume by addition of 1.32 mmol dodecane‐*d*_26_. Under the latter conditions, the yield and turnovers are comparable to the C─H alumination reaction of neat dodecane with 120 equiv of AlEt_3_ and catalyst **2** (52% yield with respect to C_12_H_26_, 36 turnovers). Because dilution did not improve the turnovers, these experiments suggest that the active site is irreversibly inhibited by the reaction mixture.

We also note that dodecane‐1,12‐diol, the product from di‐alumination and workup, was only formed in trace quantities even at relatively high conversion of dodecane. The high selectivity for mono‐alumination is surprising because, by the end of the reaction, dodecyldiethylaluminum (63%) is present in higher concentration than dodecane itself (35%) and alkanes are typically less reactive than their functionalized derivatives. We considered that a functionalized product, even at the other end of the long dodecyl chain, might inhibit and/or deactivate the zirconium catalyst. To test this hypothesis, 1‐trimethylsiloxydodecane (CH_3_C_10_H_20_CH_2_‐O‐SiMe_3_) was subjected to similar catalytic conditions in the presence of the alkoxyzirconium grafted material **2** and 120 equiv of AlEt_3_. There was no observable conversion of the substrate to HO‐CH_2_C_10_H_20_CH_2_‐O‐SiMe_3_ upon alkaline workup. To check if the catalyst was undergoing degradation or deactivation in the presence of the substrate, a 1:1 mixture of dodecane and 1‐trimethylsiloxydodecane (CH_3_C_10_H_20_CH_2_‐O‐SiMe_3_) was subjected to catalytic conversion using the catalyst **2** and 120 equiv AlEt_3_ at 150 °C for 12 h. In this experiment, dodecan‐1‐ol (12% yield with respect to dodecane, 4 turnovers) was formed while 1‐trimethylsiloxyldodecane was inert. Because the intramolecular inhibitory effect is much more significant for 1‐trimethylsiloxyldodecane, while the dilution of diethyldodecylaluminum does not increase turnovers, we conclude that stronger association of functional groups (including arenes) with zirconium sites limits the conversion and leads to catalyst decomposition. This association also inhibits difunctionalization of aliphatic hydrocarbons under the present conditions.

### Zr‐Catalyzed C─H Alumination of Methane

Because **2**‐catalyzed C─H alumination did not lead to difunctionalization of dodecane, even at high conversion, this catalyst system could also be effective for the mono‐functionalization of methane. In addition, if the catalyst inhibition was related to build‐up of dodecyldiethylaluminum, it seemed possible that diethylmethylaluminum would show less deactivation. The alumination of methane is an appealing reaction because, while AlEt_3_ is readily synthesized from aluminum, ethylene, and hydrogen in an atom‐economical pathway, AlMe_3_ preparation requires a multistep process incorporating alkali metal reduction of AlMe_2_Cl that generates NaCl and Al as by‐products.^[^ ^50^ ^]^ The direct synthesis of AlMe_3_ from AlEt_3_ and methane would be more efficient.

The supported tert‐butoxyzirconium catalyst **2**, AlEt_3_ (60 or 300 equiv), and CH_4_ (725 psi) were heated to 150 °C for 12 h in a 2 mL sealed steel reactor. The reaction mixture was then analyzed by ^1^H NMR spectroscopy using hexamethylbenzene as an internal standard. The broad ^1^H NMR singlet close to 0 ppm can be readily assigned to the AlMe group using phase edited ^13^C‐^1^H HSQC experiments. The turnover number increases following the trends seen for dodecane (60 equiv AlEt_3_: 19.7% yield based on CH_4_, 42 turnovers; 300 equiv AlEt_3_: 33.4% yield based on CH_4_, 71 TON, Equation 9). Thus, surface silylation doubled the turnovers and yield compared to that obtained from catalyst **1**, which gave 9% yield and 19 turnovers in an experiment using 60 equiv of AlEt_3_. Moreover, **1** yielded negligible amounts of methylaluminum in experiments using 150 equiv of AlEt_3_.

(9)

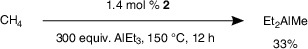




## Conclusion

The reaction of Zr(O*^t^* Bu)_4_ with SiO_2_‐Al_2_O_3–700_ yields **1**, containing 0.63 mmol of Zr·g^−1^ and leaving ∼0.03 mmol·g^−1^ of unreacted accessible ─OH groups, which is less than 5% of the original hydroxy groups in the silica–alumina. We have found that these few OH groups create a pathway for catalyst decomposition in **1**‐catalyzed C─H alumination of *n*‐dodecane, via AlEt_3_ grafting and subsequent attack by additional free AlEt_3_. This pathway limits the catalytic C─H alumination with **1** because undesired ethylsilane side‐products from silica ethylation increase as AlEt_3_ concentration increases. At the same time, the yield of dodecan‐1‐ol decreases as AlEt_3_ concentration increases.

A solution to the side reaction and catalyst decomposition is provided by capping the few residual silanols in **1** with SiMe_3_ groups by reaction with Me_3_SiCH_2_CH═CH_2_, producing **2**. **2** contains identical ≡SiO─Zr(O*^t^* Bu)_3_ sites as in **1**, yet gives more turnovers and is more selective for the catalytic C─H alumination of dodecane. In the presence of **2**, dodecan‐1‐ol is formed in up to 72% yield while the formation of unwanted ethylsilanes is suppressed. The surface silylation in **2**, and the resistance of **2** toward AlEt_3_‐mediated degradation, is supported by solid‐state ^13^C and ^29^Si NMR experiments comparing **1** and **2** before and after AlEt_3_ treatment.

Unlike **1**, catalyst **2** is not deactivated by high AlEt_3_ concentrations; however, yields are apparently limited by the dodecyldiethylaluminum product, as evidenced by the lack of activity of fresh **2** added to reaction mixtures of C_12_H_25_AlEt_2_ and AlEt_3_, the higher yields when the reaction mixture is diluted with dodecane‐*d*_26_, and the higher turnovers obtained with methane than dodecane. The differing reactivity of dodecyldiethylaluminum, triethylaluminum, and diethylmethylaluminum is possibly related to the stability of their dimers (C_12_H_25_AlEt_2_)_2_, (AlEt_3_)_2_, and (Et_2_AlMe)_2_, where the bridging methyl group in the latter limits its reactivity with the zirconium catalyst compared to (AlEt_3_)_2_ and (C_12_H_25_AlEt_2_)_2_. Because the resulting mixed alkyl(diethyl)aluminum species undergo rapid alkyl group exchange, the lowest‐boiling trialkylaluminum species can be purified by distillation (e.g., Me_3_Al), and the residual AlEt_3_ can be recovered for future use. Thus, the high selectivity of catalyst **2** for mono‐functionalization at the ends of aliphatic chains provides significant opportunity for applying this catalytic alumination in commodity‐scale synthesis.

## Supporting Information

Experimental description of the syntheses and characterization of supported zirconium catalysts **1**, **1** ^80%^, **2**, and **2^80%^**, and results of catalytic C─H alumination studies.

## Conflict of Interests

The authors declare no conflict of interest.

## Supporting information



Supporting Information

## Data Availability

The data that support the findings of this study are openly available in [DataShare] at [https://doi.org/10.25380/iastate.29437283.v1].
