# Supplementary material for: Surface Protected Organozirconium Catalyzes C─H Alumination of Saturated Hydrocarbons
Source: Angew Chem Int Ed Engl. 2025 Jul 30;64(36):e202511893. doi: 10.1002/anie.202511893 (PMC12402881; doi:10.1002/anie.202511893)
Supplement: Supplementary file 1 — Supporting Information [file ANIE-64-e202511893-s001.pdf]

Surface Protected Organozirconium Catalyzes C–H Alumination of Saturated Hydrocarbons

Sayak Banerjee,[a,b] Jessica Rodriguez,[b] Marco Mais,[b] Frédéric A. Perras,[a,b] Aaron D. Sadow*[a,b]

[1] Department of Chemistry, Iowa State University, Ames, Iowa 50011, United States

[2] U.S. DOE Ames National Laboratory, Iowa State University, Ames, Iowa 50011, United States

sadow@iastate.edu

Table of Contents.

Experimental S1-S8

DRIFTS spectra of support and catalysts S8-S12

NMR spectra of catalyst and representative catalytic reactions S13-S25

GC trace of representative catalytic reactions S26-S31

References S31-S32

Experimental.

General. All manipulations were carried out under inert conditions, either using standard Schlenk techniques or in a glovebox under a purified nitrogen atmosphere unless stated otherwise. Dry and degassed solvents were used throughout. Pentane, toluene, and benzene were sparged with nitrogen, passed through activated alumina columns, and stored under nitrogen over activated molecular sieves. Benzene-d_6 was degassed by three consecutive freeze-pump-thaw cycles, stirred over Na/K alloy, vacuum transferred, and stored over activated molecular sieves. Allyltrimethylsilane was obtained from Gelest, distilled from CaH_2, and stored over activated molecular sieves. Triethylaluminum was obtained from Fisher Scientific and used as received. **CAUTION: $AlEt_3$ is pyrophoric and appropriate air-free Schlenk-line and glovebox techniques must be applied for its handling**. During workup, all experiments containing $AlEt_3$ were diluted with toluene and then carefully quenched at 0 °C under a stream of dry oxygen. Silica-alumina (Grade 135) of surface area 475 m^2g^{-1} was purchased from SigmaAldrich, calcined at 600

°C for 12 h, and partially dehydroxylated at 700 °C for 12 h (ramping rate 5 °C·min^{-1}) under vacuum (3.8×10^{-7} torr) (identified as SiO_2-$Al_2O_{3\text{-}700}$ in this work). Titration of the surface hydroxyl groups in this material with $Mg(CH_2Ph)_2$ revealed a surface OH loading of 0.66 ± 0.02 mmol·g^{-1} (0.83 OH/nm^2).

Solution phase 1D ^{1}H NMR and ^{13}C{^{1}H} NMR spectra and 2D ^{1}H-^{1}H COSY and ^{1}H-^{13}C HSQC NMR spectra were acquired on a Bruker NEO 400 MHz and Bruker Avance 600 MHz Spectrometers.

Fourier transform infrared (FT-IR) spectra and diffuse reflectance infrared Fourier Transform spectra (DRIFTS) were recorded on a Bruker VERTEX 80 IR spectrometer. Samples for IR configured for transmission mode were diluted with anhydrous KBr, finely ground using a mortar and pestle, and pressed into a transparent pellet using a hydraulic press. DRIFTS were collected using a Harrick Praying Mantis accessory in a sealed, ambient-pressure chamber consisting of a dome with ZnSe windows.

Gas chromatography-mass spectrometry (GC-MS) was performed on an Agilent 7890 A GC system equipped with an Agilent 5975 C MSD mass spectrometer to analyze low molecular weight products. An Agilent DuraBond DB-5HT (30 m) capillary column was used for the separation of the compounds. Samples were prepared by dissolving 0.1 mL of the oil products in dichloromethane containing mesitylene (72.5 mM) as an internal standard. A standard calibration plot using a series of known concentrations of authentic samples (namely, n-dodecane and n-dodecanol) and the stock solution of mesitylene in methylene chloride (0.5 mL) was prepared. The oil product from the catalytic reactions were analyzed based on the integrated peak areas of dodecane and dodecanol vs mesitylene using GC-MS, and then concentrations were calculated from the calibration plot.

Gas chromatography-flame ionization detection was performed on Agilent Technologies G1530A using a J&W GasPro (0.32 mm × 15 m) capillary column. A standard calibration plot of peak area vs. pressures of methane (in mmHg) was constructed. Manual injection of 100 μL of the sample, vacuum transferred from the reaction mixture into a 3L round bottom flask fitted with a resealable Teflon tapped Schlenk adapter, was conducted using a 250 μL Hamilton Gastight#1725 sample lock syringe. The areas of ethane and propene were correlated to the area of methane based on the effective carbon number (ECN) equation.[1] The concentration (in moles) were calculated using the ideal gas law.

Inductively Coupled Plasma-Optical Emission Spectroscopy (ICP-OES) was performed on an Agilent 5800 ICP-OES to measure the zirconium and aluminum content present in the catalytic materials. The samples (2.0 mg to 6.0 mg each) were digested for 24 h in aqueous HF and aqua-regia (0.18% and 5% respectively).

Solid-State NMR Spectroscopy. Dynamic nuclear polarization (DNP)-enhanced ^{13}C and ^{29}Si cross-polarization magic-angle spinning (CPMAS) NMR spectra were acquired using a Bruker AVANCE III MAS-DNP NMR spectrometer equipped with a 400 MHz NMR magnet, a 264 GHz gyrotron microwave source, and a triple-resonance low-temperature MAS probe tuned to ^{1}H-^{13}C-^{29}Si. In all cases, relaxation delays were set to 2.6 s, optimized using a saturation recovery experiment, and the ^{1}H excitation pulse used a 100 kHz radiofrequency power. ^{29}Si CP was accomplished using a 5 ms contact time while a 200 μs contact time was used for ^{13}C, to minimize the intensity of the CPMAS signal from the solvent used: 1,1,2,2-tetrachloroethane-d_2.[2,3] All samples were prepared in a glovebox where they were packed into 3.2 mm sapphire rotors and impregnated with a 16 mM dry solution of the TEKPol polarizing agent.[4] Samples were then transported in a sealed vial to a pre-cooled probe and quickly inserted into the nitrogen atmosphere of the system. Spectra were typically acquired in 1024 scans.

	**Elements (mmol/g)**		
**Materials**	**Zr**	**Al**	**Si**
SiO_2-$Al_2O_{3\text{-}700}$	n.a.	0.31 ± 0.02	2.37 ± 0.04
$Zr(O^tBu)_3$@SiO_2-$Al_2O_{3\text{-}700}$ (**1**)	0.63 ± 0.03	0.29 ± 0.04	2.36 ± 0.03
$Zr(O^tBu)_3^{80\%}$@SiO_2-$Al_2O_{3\text{-}700}$ (**1$^{80\%}$**)	0.46 ± 0.02	0.30 ± 0.02	2.40 ± 0.03
$Zr(O^tBu)_3$/$SiMe_3$@SiO_2-$Al_2O_{3\text{-}700}$ (**2**)	0.59 ± 0.03	0.29 ± 0.03	2.35 ± 0.03
$Zr(O^tBu)_3^{80\%}$/$SiMe_3$@SiO_2-$Al_2O_{3\text{-}700}$ (**2$^{80\%}$**)	0.44 ± 0.02	0.29 ± 0.02	2.33 ± 0.03
**1** + 5 $AlEt_3$	0.58 ± 0.03	0.37 ± 0.04	2.38 ± 0.02
**2** + $AlEt_3$	0.57 ± 0.02	0.31 ± 0.02	2.38 ± 0.04

Silylation-protection of silica-alumina ($SiMe_3$@SiO_2-$Al_2O_{3\text{-}700}$) with propene quantification. A 10 mL glass tube equipped with a Teflon-coated magnetic stir bar was charged with SiO_2-$Al_2O_{3\text{-}700}$ (0.500 g; 0.33 mmol OH), trimethylallylsilane (0.149 g, 1.31 mmol; 4.0 equiv), and toluene (0.2 mL). The tube was connected to a glass Teflon-valved vacuum adapter using an UltraTorr fitting, and the vacuum adaptor was connected to a Schlenk line. The reaction

mixture was then frozen at –196 °C, the headspace was completely evacuated, and the reactor was sealed. The reactor vessel was allowed to slowly warm to room temperature and then was heated at 150 °C with rapid stirring for 12 h. The reaction mixture was cooled, and all the volatiles were transferred into a 3L round bottom flask equipped with a Schlenk adapter with re-sealable Teflon tap. The SiO_2-$Al_2O_{3\text{-}700}$ in the reactor was then resubjected to reaction with trimethylallylsilane in the same manner two additional times to ensure complete silylation of the silanol groups. Each time, the volatiles were collected in the 3L round-bottom flask (via vacuum transfer). The propene gas produced in the grafting reaction, now contained in the 3L round-bottom flask, was injected into a GC-FID for quantification (0.65 ± 0.03 mmol·propene g^{-1}). This value matches the amount of OH's present in the SiO_2-$Al_2O_{3\text{-}700}$, suggesting all silanols were capped by trimethylsilyl groups. This synthesis can be performed without propene quantification in a re-sealable glass pressure vessel at ambient pressure. The residual surface OHs were titrated using $Mg(CH_2Ph)_2$, and the resulting toluene was quantified using $Si(SiMe_3)_4$ as an internal standard (~0.004 mmol OH·g^{-1}).

$Zr(O^tBu)_3$@SiO_2-$Al_2O_{3\text{-}700}$ (1). Material **1** was prepared following the protocol in reference [5]. The synthesis is given here for comparison to new experiments in this work. Excess $Zr(O^tBu)_4$ (0.377 g, 0.983 mmol, 1.5 equiv) was dissolved in pentane (10 mL). This solution was added to a suspension of SiO_2-$Al_2O_{3\text{-}700}$ (1.00 g, 0.66 mmol OH) in pentane (15 mL). The reaction mixture was stirred for 12 h. The suspension was allowed to settle, and the supernatant was decanted. The residual solid was washed pentane (3×7 mL) and then dried overnight in vacuo to afford a white powder (0.95 g). This material contains 2.9 ± 0.2 O^tBu groups per Zr center as determined by the titration of $Zr(O^tBu)_3$@SiO_2-$Al_2O_{3\text{-}700}$ with HCOOH in benzene-d_6 in the presence of $Si(SiMe_3)_4$ as an internal standard (~1.77 mmol O^tBu·g^{-1}). The amount of ^tBuOH produced in the grafting reaction was quantified using ^1H NMR spectroscopy (0.59 ± 0.05 mmol ^tBuOH·g^{-1}), and this amount was compared to the zirconium loading determined by ICP-OES (0.63 ± 0.03 mmol of Zr/g). The OH groups in catalyst **1** were titrated using $Mg(CH_2Ph)_2$ to reveal 0.041 mmol OH/g from residual silanol groups in $Zr(O^tBu)_3$@SiO_2-$Al_2O_{3\text{-}700}$ after $Zr(O^tBu)_4$ grafting. This value agrees well with the expected residual OH (0.044 mmol/g) based on initial OH loading in SiO_2-$Al_2O_{3\text{-}700}$ and the loading of Zr in $Zr(O^tBu)_3$@SiO_2-$Al_2O_{3\text{-}700}$. The DRIFTS is given below.

$Zr(O^tBu)_3^{80\%}$@SiO_2-$Al_2O_{3\text{-}700}$ ($1^{80\%}$). A solution of $Zr(O^tBu)_4$ (0.201 g, 0.524 mmol) in pentane (10 mL) was added to a suspension of SiO_2-$Al_2O_{3\text{-}700}$ (1.00 g, 0.66 mmol OH) in pentane

(15 mL). The reaction mixture was stirred for 12 h. The suspension was allowed to settle, and the supernatant was decanted. The residual solid was washed with benzene (3×7 mL), pentane (2×7 mL), and then dried overnight in vacuo to afford a white powder (0.96 g). The zirconium loading determined by ICP-OES (0.46 ± 0.02 mmol of Zr/g). Titration of OH groups in catalyst $\mathbf{1}^{80\%}$ with $Mg(CH_2Ph)_2$ indicated that the material contained 0.114 mmol OH/g. This value agrees well with the expected residual OH (0.113 mmol/g) based on initial OH loading in SiO_2-$Al_2O_{3\text{-}700}$ and the loading of Zr in $Zr(O^tBu)_3$@SiO_2-$Al_2O_{3\text{-}700}$. The DRIFTS is given below.

$Zr(O^tBu)_3$/$SiMe_3$@SiO_2-$Al_2O_{3\text{-}700}$ (2). A 10 mL glass tube was charged with catalyst **1** (1.0 g, 0.63 mmol Zr) and a Teflon-coated magnetic stir bar. Trimethylallylsilane (18.7 mg, 0.164 mmol, 4 equiv) and toluene (0.5 mL) were added. The reaction mixture was cooled to –196 °C, the headspace was evacuated, and the reactor was sealed. The reaction mixture was allowed to warm to room temperature and then was heated to 150 °C with rapid stirring for 12 h. The reaction mixture was then allowed to cool to room temperature, and all volatiles were vacuum transferred into a 3 L round bottom flask fitted with a resealable Schlenk adapter for propene quantification. Injection of the gas into GC-FID for analysis revealed 0.07 ± 0.03 mmol·g^{-1} were formed, in good agreement with capping the residual OH groups in **1**. The solid remaining in the reactor was a light cream-colored powder (0.96 g). Analysis of Zr by ICP-OES reveals 0.59 ± 0.03 mmol·g^{-1} in **2**. In addition, titration of **2** with formic acid released 1.65 mmol of ^tBuOH·g^{-1}, corresponding to 2.8 O^tBu per Zr. The DRIFTS is given below.

$Zr(O^tBu)_3^{80\%}$/$SiMe_3$@SiO_2-$Al_2O_{3\text{-}700}$ ($\mathbf{2}^{80\%}$). A 10 mL glass tube was charged with material $\mathbf{1}^{80\%}$ (1.0 g) and a Teflon-coated magnetic stir bar. Trimethylallylsilane (52.1 mg, 0.456 mmol, 4 equiv) and toluene (0.5 mL) were added. The reaction mixture was cooled to –196 °C, the headspace was evacuated, and the reactor was sealed. The reaction mixture was allowed to warm to room temperature and then was heated to 150 °C with rapid stirring for 12 h. The reaction mixture was then allowed to cool to room temperature. All volatiles were vacuum transferred into a 3 L round bottom flask fitted with a resealable Schlenk adapter for gas (propene) quantification by GC-FID, which revealed 0.112 mmol propene·g^{-1} were formed), in good agreement with capping the residual OH groups in $\mathbf{1}^{80\%}$. The procedure afforded a light cream-colored powder (0.96 g). Analysis of Zr by ICP-OES reveals 0.44 ± 0.02 mmol·g^{-1} in $\mathbf{2}^{80\%}$. The DRIFTS is given below.

$Zr(O^tBu)_3$@SiO_2-$Al_2O_{3\text{-}700}$ + 5 $AlEt_3$. Material **1** (0.011 mmol Zr) and excess $AlEt_3$ (0.055 mmol, 6.3 mg, 5 equiv) were allowed to react at room temperature in benzene-d_6 over 4 h, at which point the mixture was filtered, and the solution was analyzed. The residual solid was dried and analyzed. All soluble alkyl and alkyloxyaluminum species were quantified by solution-phase NMR spectroscopy by comparison of ^{1}H NMR integrals to a $Si(SiMe_3)_4$ internal standard. ^{1}H NMR signals at 0.14 ppm and 1.22 ppm, attributed to the $Al\mathit{CH_2}CH_3$ and $AlCH_2\mathit{CH_3}$, were distinct from the resonance of the unreacted $AlEt_3$, which appeared at 0.3 ppm (q) and 1.1 ppm (t) for $Al\mathit{CH_2}CH_3$ and $AlCH_2\mathit{CH_3}$, respectively. A singlet at 0.8 ppm corresponded to C_2H_6. A singlet at 1.1 ppm was assigned to the $AlOC\mathit{Me_3}$ of $AlEt_2O^tBu$ based on a multiplicity-edited ^{1}H-^{13}C HSQC experiment. Integration of ^{1}H NMR signals revealed 0.058 mmol C_2H_6/g was formed and 0.006 mmol O^tBu had been transferred to Al, corresponding to only 18% of the total O^tBu groups present. ICP-OES: 0.58 ± 0.03 mmol Zr·g^{-1}, 0.37 ± 0.04 mmol Al·g^{-1}.

$Zr(O^tBu)_3/SiMe_3$@SiO_2-$Al_2O_{3\text{-}700}$ + $AlEt_3$. The material **2** (0.011 mmol Zr) was allowed to react with excess $AlEt_3$ (5 or 60 equiv) at room temperature over 4 h in benzene-d_6 to provide $AlEt_2(O^tBu)$ and ≡SiO–$Zr(O^tBu)_2Et$ surface species. Soluble alkyl and alkyloxyaluminum species were detected and quantified by solution-phase NMR spectroscopy by comparison of ^{1}H NMR integrals to a $Si(SiMe_3)_4$ internal standard, following assignments above. ICP-OES: 0.58 ± 0.02 mmol Zr·g^{-1}, 0.32 ± 0.02 mmol Al·g^{-1}.

Representative Example of 2-Catalyzed Alumination of n-Dodecane. Catalyst **2** (0.032 g, 0.019 mmol Zr) and $AlEt_3$ (0.434 g, 3.8 mmol, 200 equiv) were mixed using a glass-coated stir bar for ca. 2 mins in a 5 mL pressure vessel to yield a light yellow solid. n-Dodecane (0.225 g, 1.321 mmol) was added to the reactor vessel, which was then sealed. The reaction mixture was allowed to react for 12 h at 150 °C with rapid stirring. The reaction vessel was cooled, transferred into a glovebox, toluene (3 mL) was added, and the Teflon cap was replaced with a septum. The reactor was taken out of the glovebox and cooled to 0 °C, and the aluminum species were quenched by flowing a stream of dry oxygen through the vessel overnight. The quenched reaction mixture was then stirred with 15% aqueous NaOH (1.0 mL, 1:1 with respect to Al) to precipitate Al/Na salts and filtered. The residual solids were extracted with toluene (2×3 mL). The residue was further extracted with boiling toluene, and the extracted portions were combined. The organic species were dried over Na_2SO_4. The volatile species were evaporated under reduced pressure at 50 °C to give a yellow oil. The oil product was characterized by NMR and GC-MS, giving 65%

(0.863 mmol) conversion of dodecane and 63% yield (0.836 mmol) of dodecanol, corresponding to 44 turnovers.

Table S1. 1-Catalyzed Alumination of n-Dodecane[a]

$AlEt_3$ mmol (equiv)	n-$C_{12}H_{26}$ mmol	% Conversion	*n*-$C_{12}H_{25}OH$ yield, mmol	% yield, vs n-$C_{12}H_{26}$	% yield, vs $AlEt_3$	TON
0.57 (30)	0.998	24	0.289	22	51	15
1.14 (60)	0.922	30	0.367	28	32	19
1.71 (90)	1.139	14	0.145	11	8	8
2.28 (120)	1.22	8	0.077	6	3	4
2.85 (150)	1.263	4	0.042	3	1	2

[a] **1** (0.032 g, 0.019 mmol Zr), $AlEt_3$ (n equiv), n-$C_{12}H_{26}$ (1.321 mmol), 150 °C, 12 h.

Table S2. 2-Catalyzed Alumination of n-Dodecane[a]

$AlEt_3$ mmol (equiv)	n-$C_{12}H_{26}$ mmol	% Conversion	*n*-$C_{12}H_{25}OH$ yield, mmol	% yield, vs n-$C_{12}H_{26}$	% yield, vs $AlEt_3$	TON
0.57 (30)	0.989	25.1	0.308	23.3	53	16
1.14 (60)	0.679	48.6	0.623	47.2	54	33
1.71 (90)	0.607	54.0	0.687	52.0	40	36
2.28 (120)	0.584	55.8	0.716	54.2	31	37
2.85 (150)	0.54	59.1	0.752	56.9	26	40
4.00 (200)	0.458	65.3	0.836	63.3	21	44

[a] **2** (0.032 g, 0.019 mmol Zr), $AlEt_3$ (n equiv), n-$C_{12}H_{26}$ (1.321 mmol), 150 °C, 12 h.

Representative Example of 2-Catalyzed Methane Alumination. These reactions were done in 2 mL steel reactors constructed from 2.5 cm (length) steel tubing (1/2” OD, 0.049” wall thickness, Part# SS-T8-S-049-20) sealed at one end with a 1/2” sized compression fitting cap (Part# SS-810-C). Catalyst **2** (0.032 g, 0.019 mmol Zr) and $AlEt_3$ (0.434 g, 3.8 mmol, 300 equiv) were added and mixed using a glass-coated magnetic stir bar for ca. 2 mins. A Swagelok ball valve (1/8”, Part# SS-41G-S2) with a 1/8” compression fitting on one end and a reducing union (1/8” to 1/2”, Part# SS-810-6-2) was attached to the open end of the tube. The valve was closed, sealing the reactor. The reactor was then attached via the 1/8” compression fitting to a 1/8” steel manifold connected to a methane cylinder and a vacuum pump, which can be switched using a three-way valve. The methane manifold was cycled with methane and vacuum 3×, and then it was pressurized

to 50 bar (750 psi). The ball valve was opened to pressurize the reactor vessel with methane. The ball valve was closed to seal the reactor. The reaction mixture was allowed to heat at 150 °C for 12 h. The reaction vessel was cooled, transferred into a glovebox. The pressure was released slowly, and a solution of C_6Me_6 standard in benzene-d_6 (1 mL) was added to the reactor. The products were characterized and quantified by ^{1}H NMR spectroscopy, giving 33.4% MeAl (1.35 mmol of methylaluminum, turnover of 71).

DRIFTS spectra of supports and catalysts

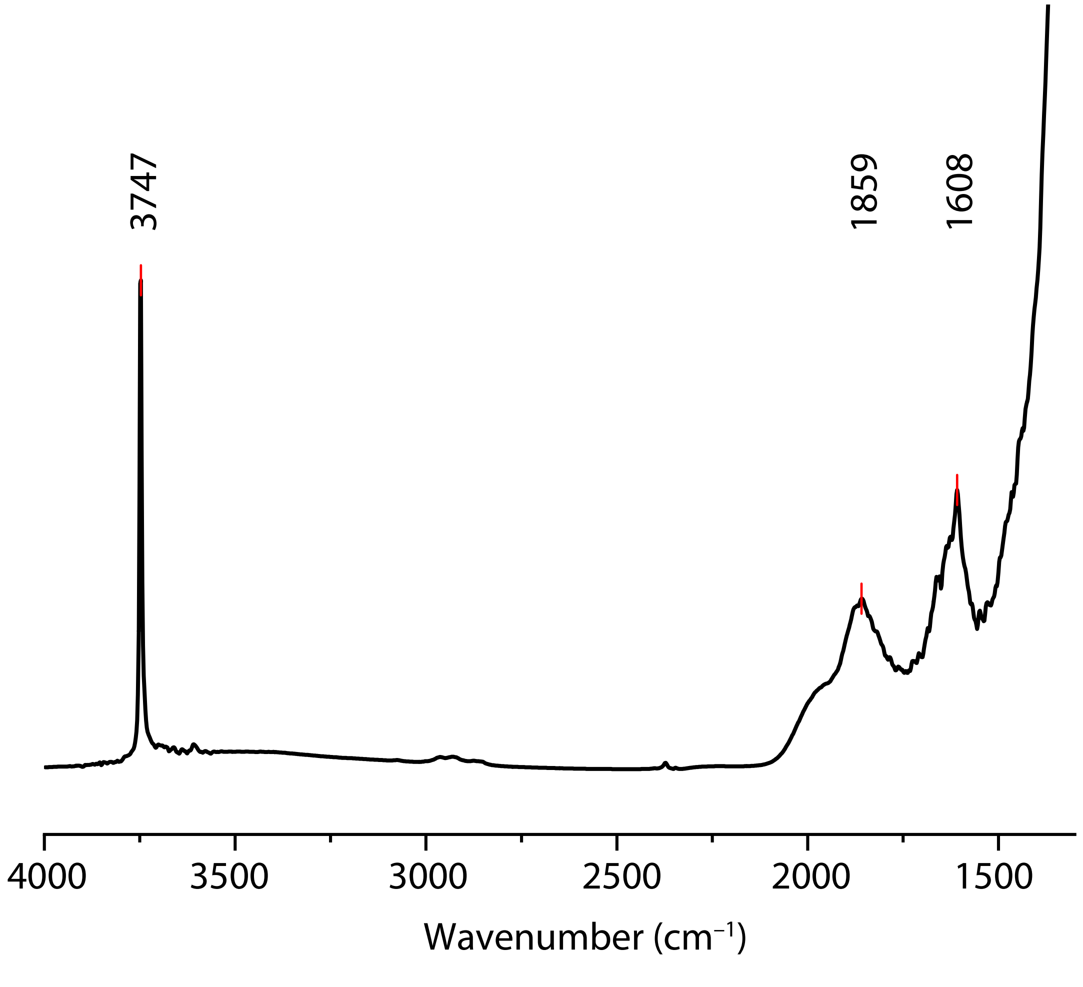

Figure S1. DRIFTS spectrum of SiO_2-$Al_2O_{3\text{-}700}$ calcined and partially dehydroxylated at 700 °C.

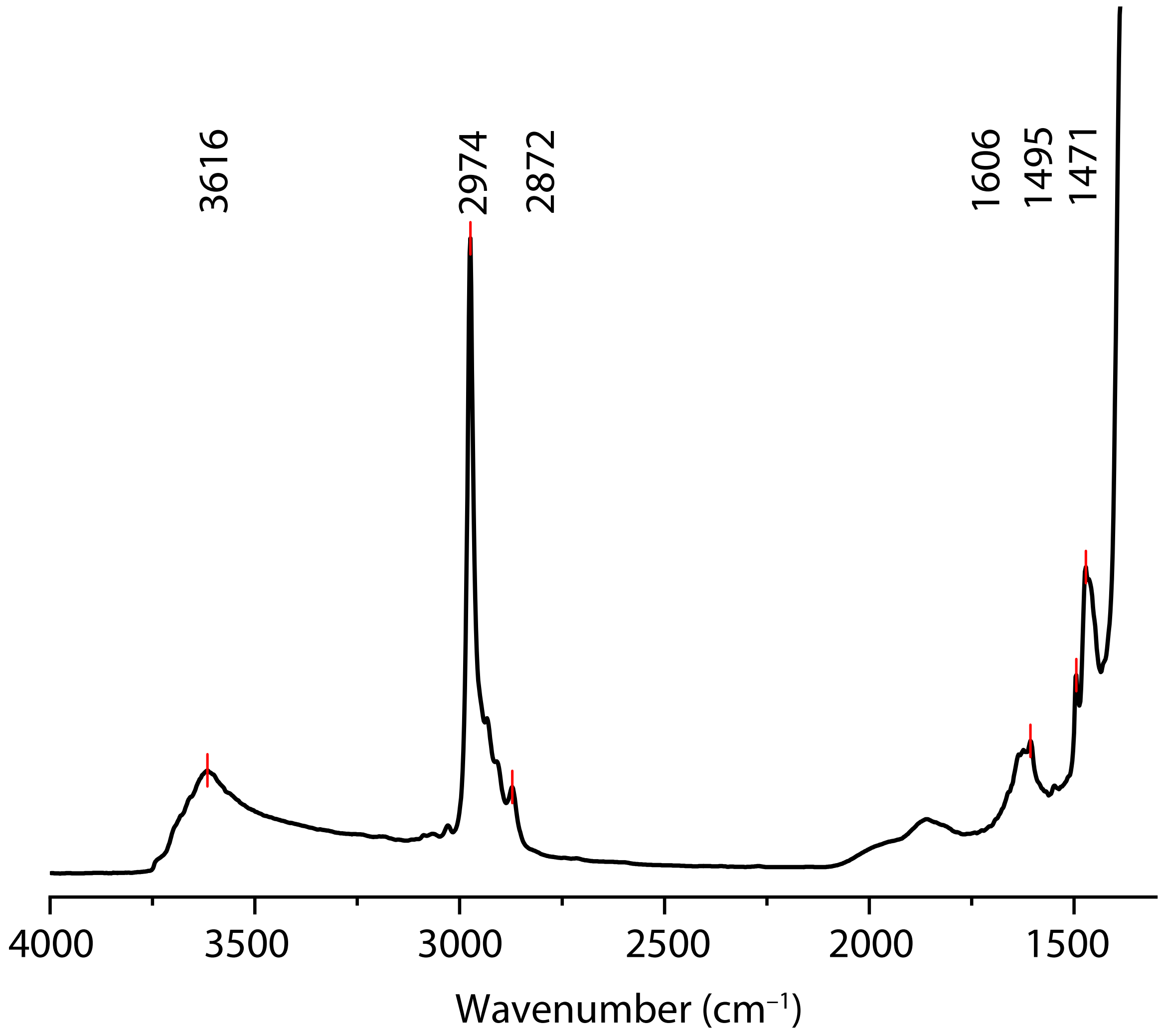

Figure S2. DRIFTS spectrum of $Zr(O^tBu)_3@SiO_2\text{-}Al_2O_{3\text{-}700}$ (**1**).

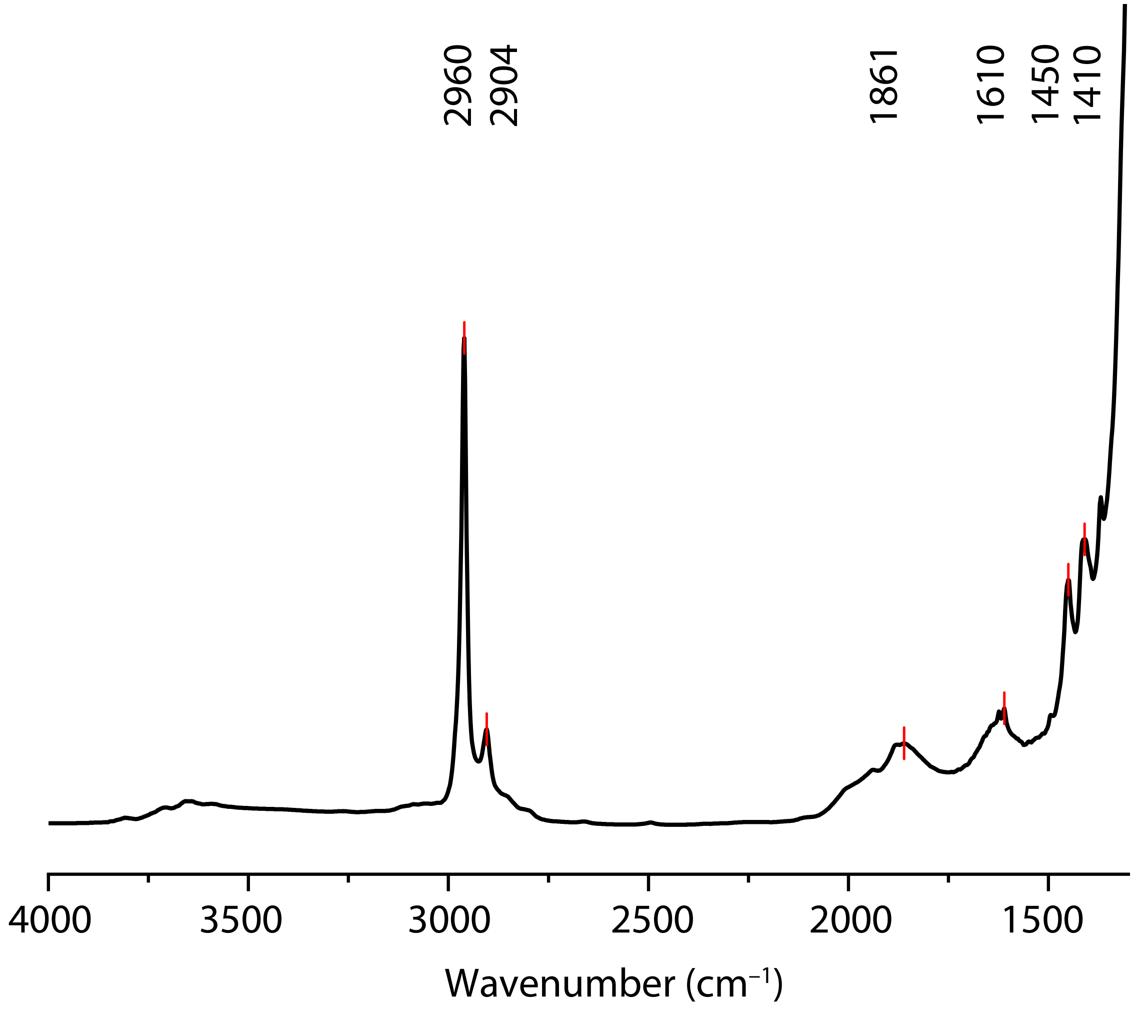

Figure S3. DRIFTS spectrum of $Zr(O^tBu)_3/SiMe_3@SiO_2\text{-}Al_2O_{3\text{-}700}$ (**2**).

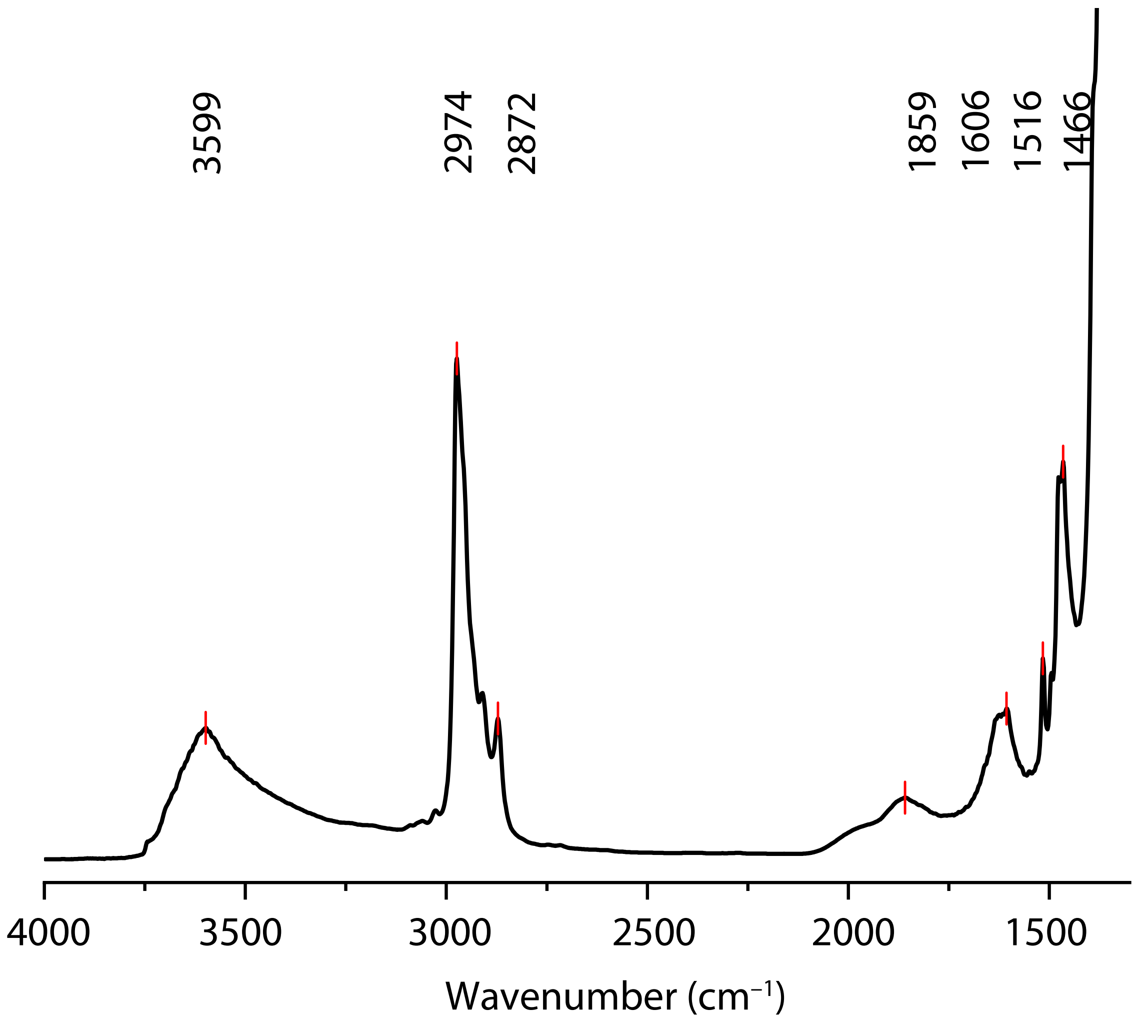

Figure S4. DRIFTS spectrum of Zr(O^{t}Bu)$_3$$^{80\%}$@SiO$_2$-Al$_2O_{3\text{-}700}$ (**1$^{80\%}$**).

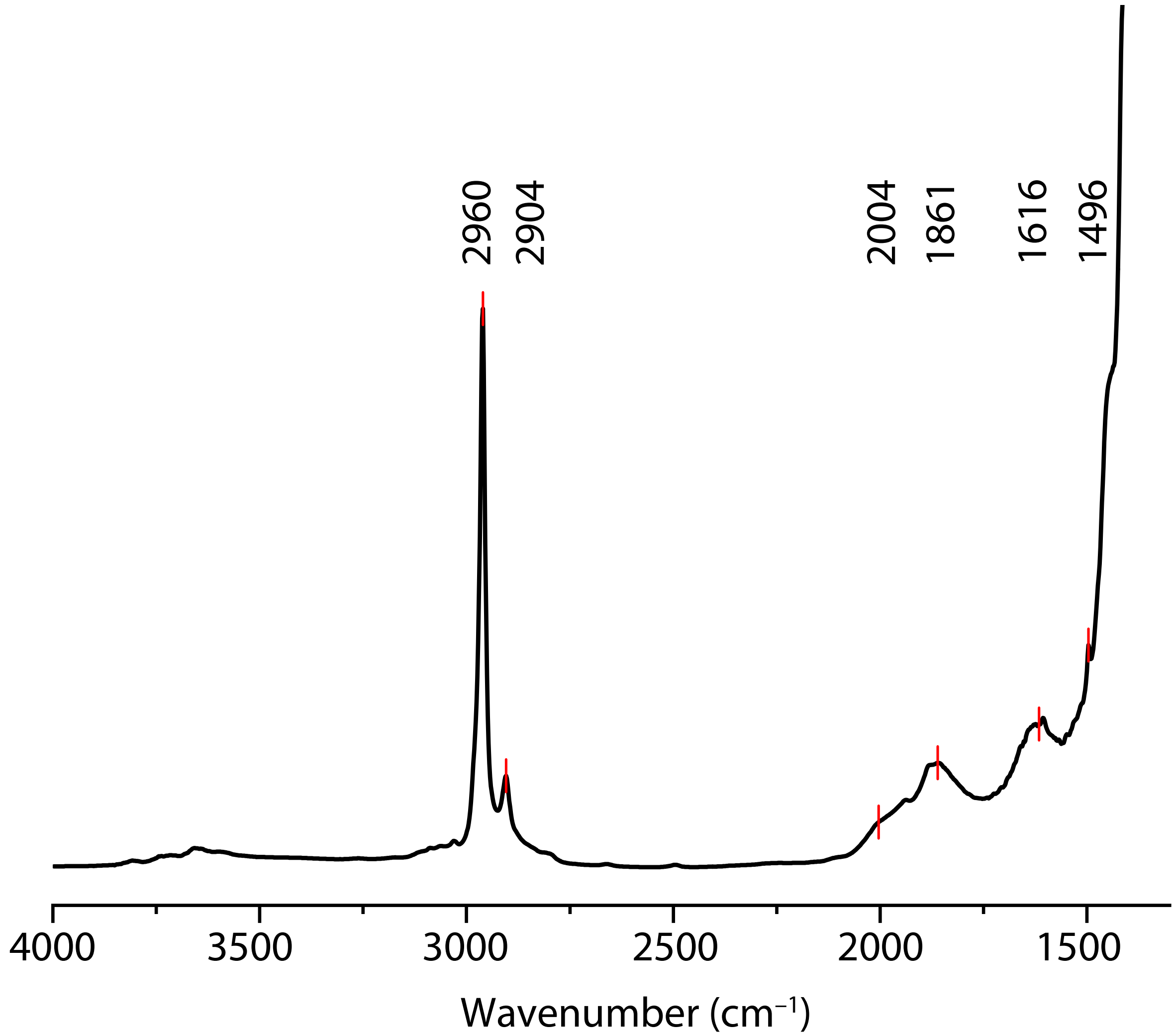

Figure S5. DRIFTS spectrum of $Zr(O^tBu)_3{}^{80\%}/SiMe_3{}^{20\%}@SiO_2\text{-}Al_2O_{3\text{-}700}$ (**$2^{80\%}$**).

NMR spectra of catalyst and representative catalytic reactions

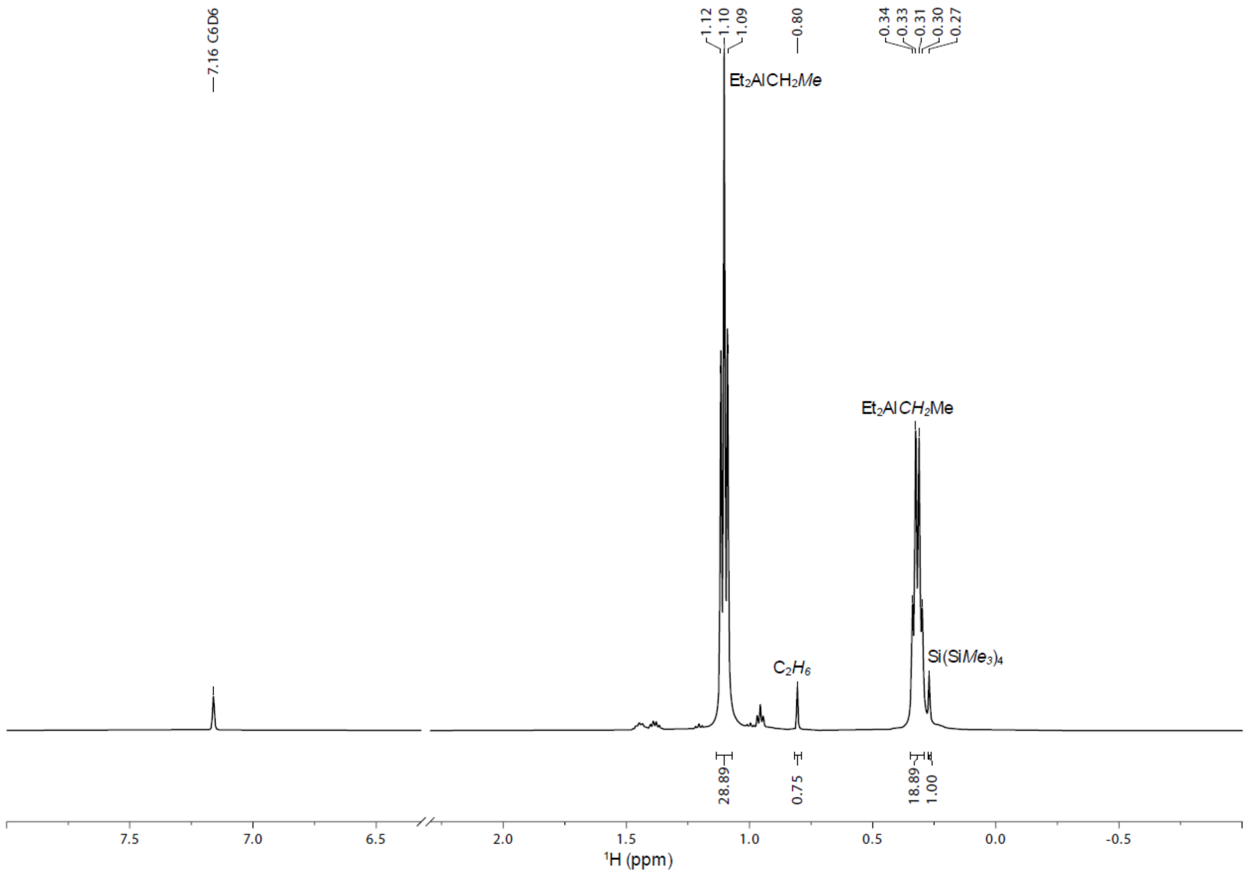

Figure S6. ^{1}H NMR (600 MHz, benzene-d_6) spectrum of the reaction of **SiO_2-$Al_2O_{3\text{-}700}$** (20 mg) with excess $AlEt_3$ (43.1 mg, 0.36 mmol) in presence of $Si(SiMe_3)_4$ (3.05 mM, 1 mL; 0.27 ppm) as internal standard. Ethane was observed as the signal at 0.8 ppm.

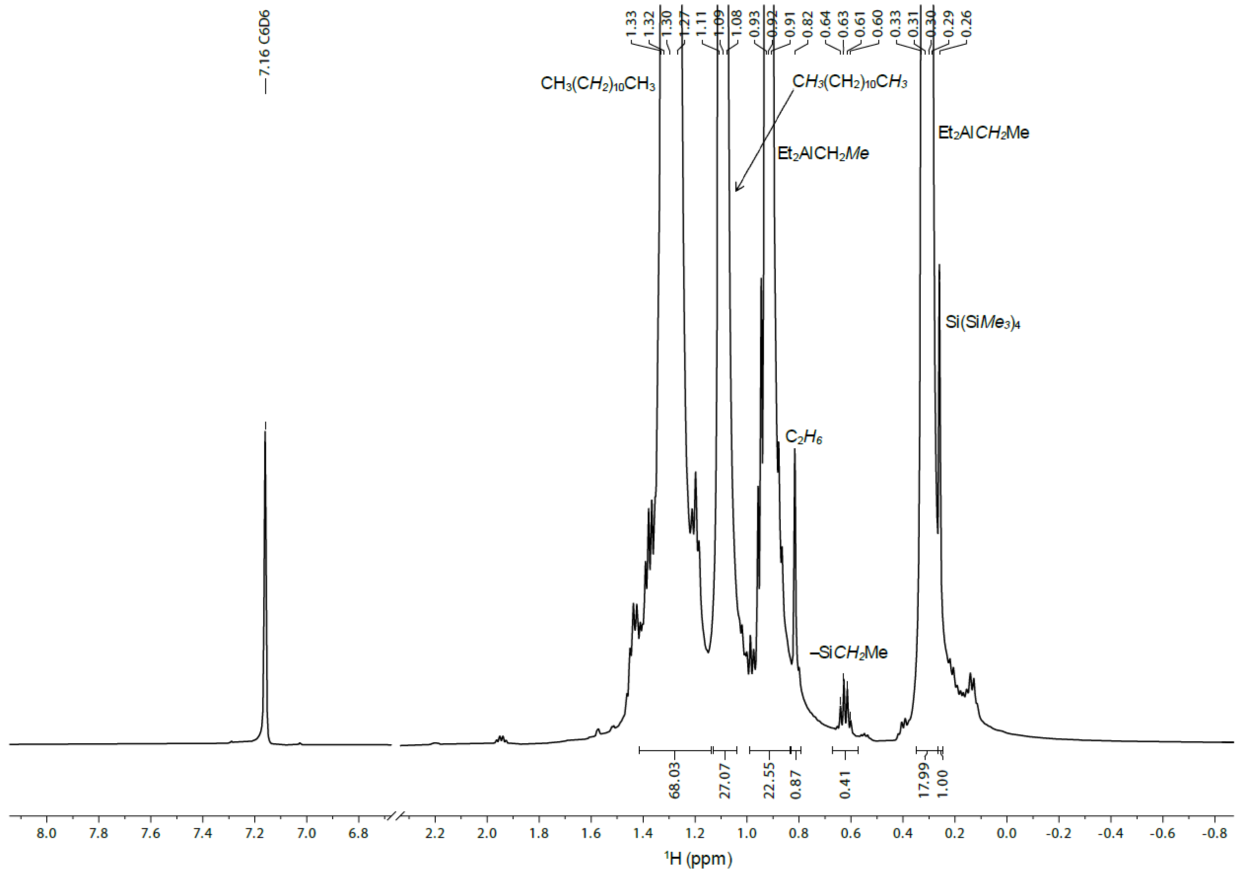

Figure S7. ^{1}H NMR (600 MHz, benzene-d_6) spectrum of the reaction of **SiO_2-Al_2O_{3-700}** (40 mg) with excess $AlEt_3$ (1.26 mmol, 144.7 mg, 48 equiv) and dodecane (2.62 mmol 446 mg) at 150 °C for 12 h in presence of $Si(SiMe_3)_4$ (3.05 mM, 1 mL) as internal standard. Ethane and ethyl silyl species were observed as signals at 0.8 and 0.6 ppm.

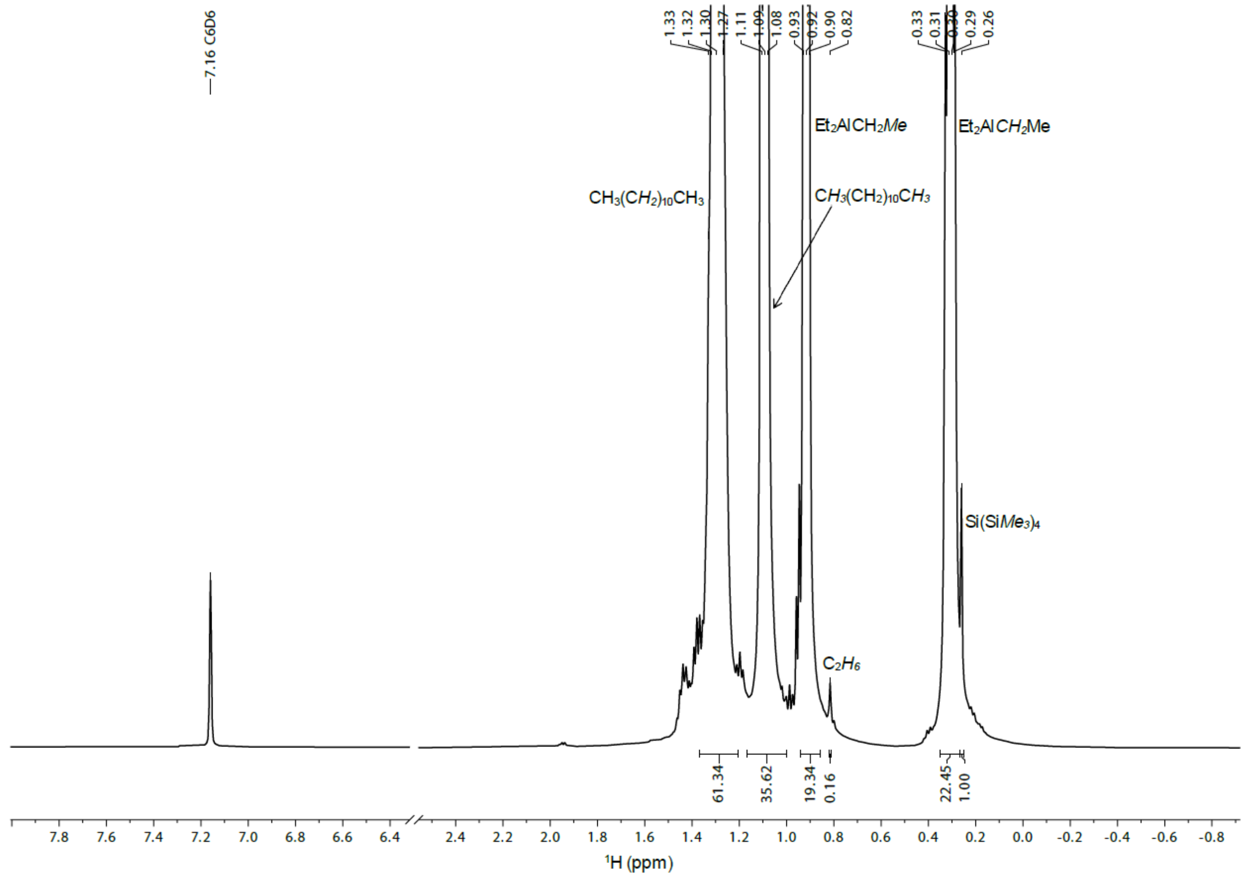

Figure S8. ^{1}H NMR (600 MHz, benzene-*d*$_6$) spectrum of the reaction of **$SiMe_3$@SiO_2-$Al_2O_{3\text{-}700}$** (40 mg) with excess $AlEt_3$ (1.26 mmol, 144.7 mg, 48 equiv) and dodecane (2.62 mmol 446 mg) at 150 °C in presence of $Si(SiMe_3)_4$ (3.05 mM, 1.0 mL) as an internal standard. The ethane at 0.8 ppm is greatly diminished while ethyl silyl species peak centered at 0.6 ppm (previous case) not observed.

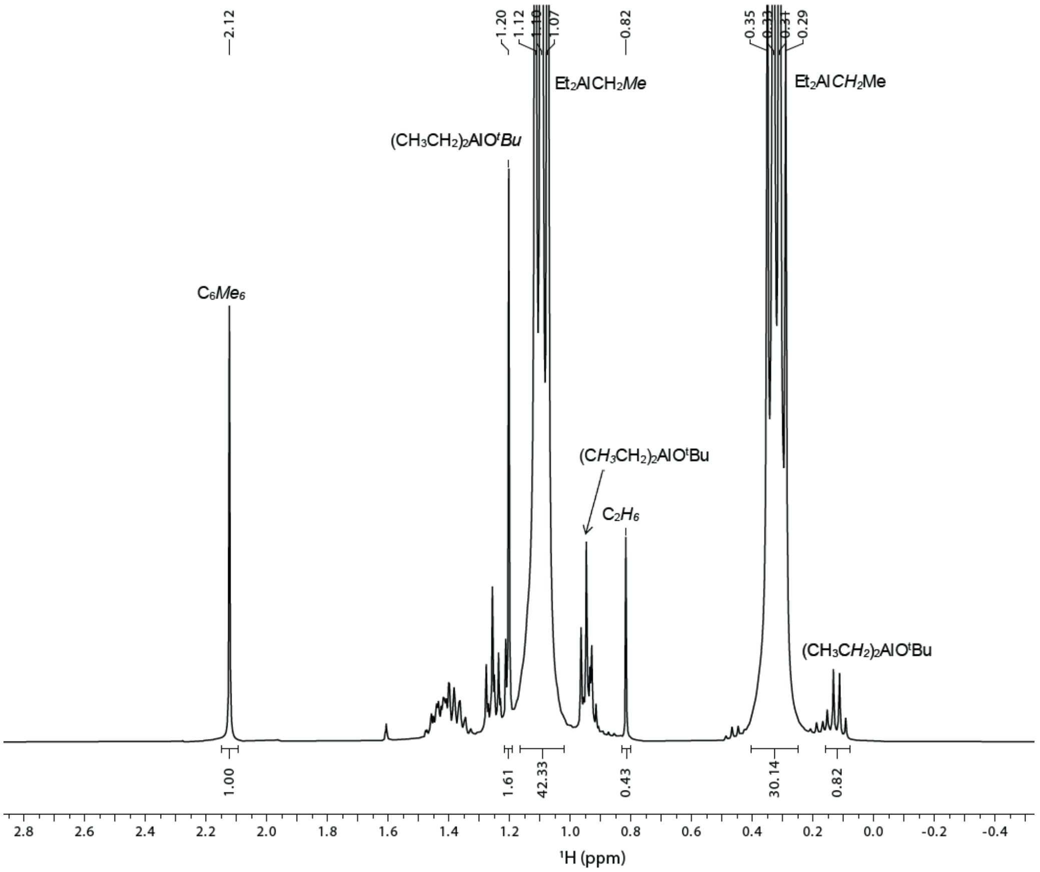

Figure S9. ^{1}H NMR (400 MHz, benzene-d_6) spectrum of the reaction of **1** (40 mg, 0.025 mmol Zr) and excess $AlEt_3$ (1.512 mmol, 60 equiv, with respect to Zr, 172.6 mg) at room temperature in presence of C_6Me_6 (2.4 mg) as an internal standard. The singlet at 1.20 ppm is the *tert*-butoxy group of Et_2AlO^tBu (0.047 mmol), corresponding to 66% of the O^tBu moieties in material **1** that were transferred to Al.

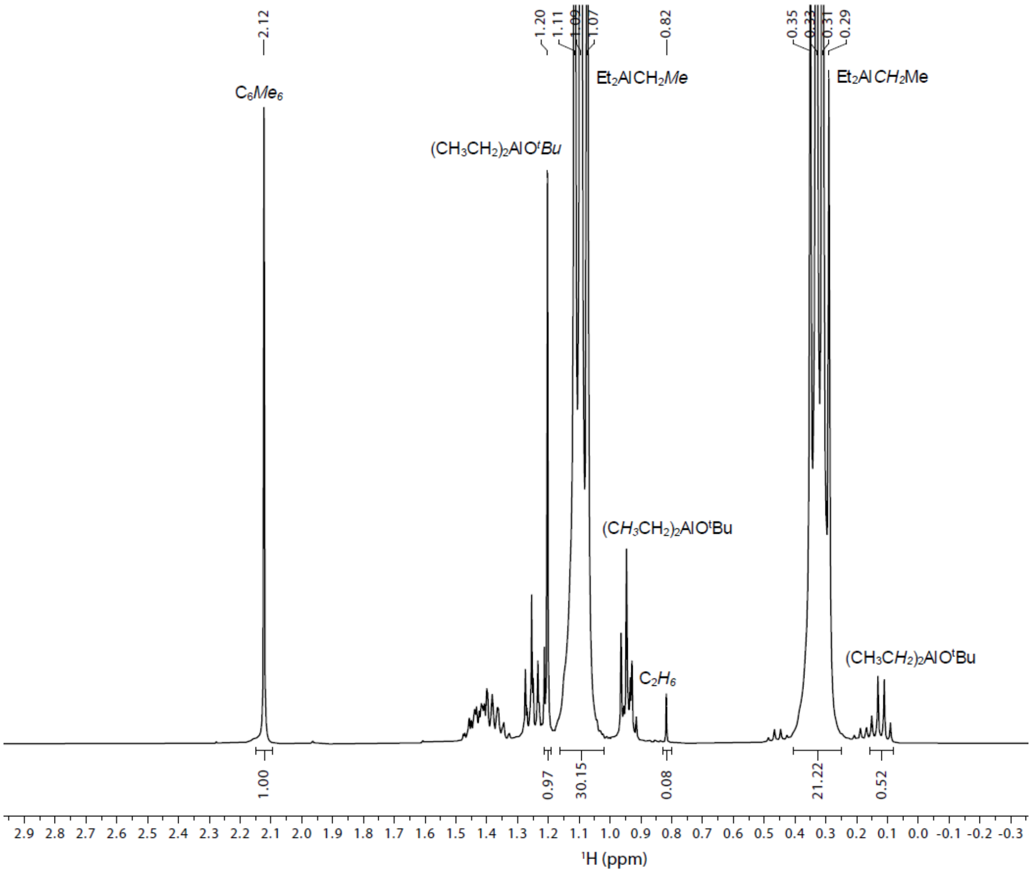

Figure S10. ^{1}H NMR (400 MHz, benzene-d_6) spectrum of the reaction of **2** (40 mg, 0.0236 mmol Zr) and excess $AlEt_3$ (1.42 mmol, 60 equiv, with respect to Zr, 161.7 mg) at room temperature in presence of C_6Me_6 (4 mg) as an internal standard. The singlet at 1.20 ppm is the $Et_2AlO^t\underline{Bu}$ (0.047 mmol), corresponding to 66% of the O^tBu moieties in material **2** that were transferred to Al.

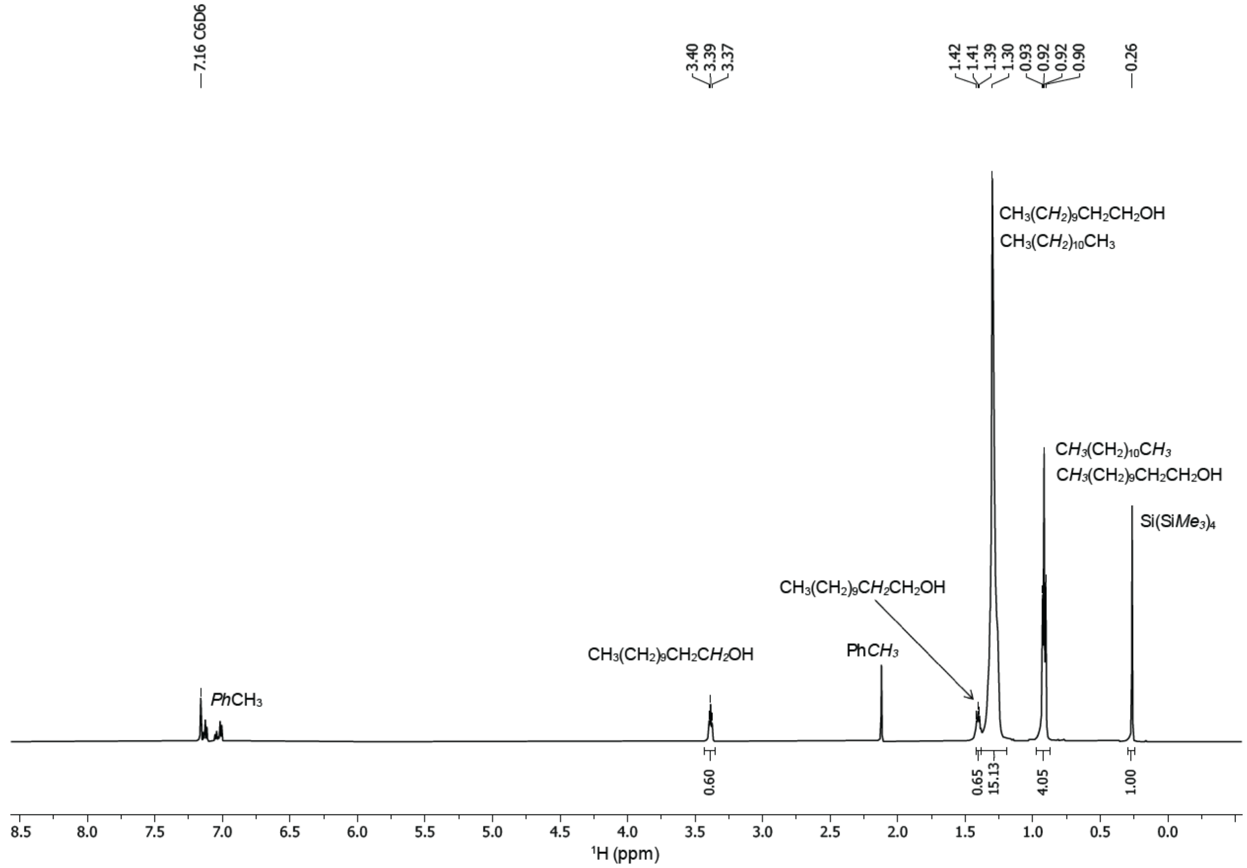

Figure S11. ^{1}H NMR (600 MHz, benzene-d_6) spectrum of the dodecan-1-ol and *n*-dodecane mixture obtained from the **1**-catalyzed (**1**: 30 mg, 0.019 mmol Zr) reaction of *n*-dodecane (1.3 mmol) and $AlEt_3$ (1.14 mmol; 60 equiv, with respect to Zr, 130.1 mg) for 12 h at 150 °C. $Si(SiMe_3)_4$ (25 mM, 1mL) was used as an internal standard. Peaks in the aromatic region and the singlet at 2.1 ppm are due to some residual toluene from the reaction workup.

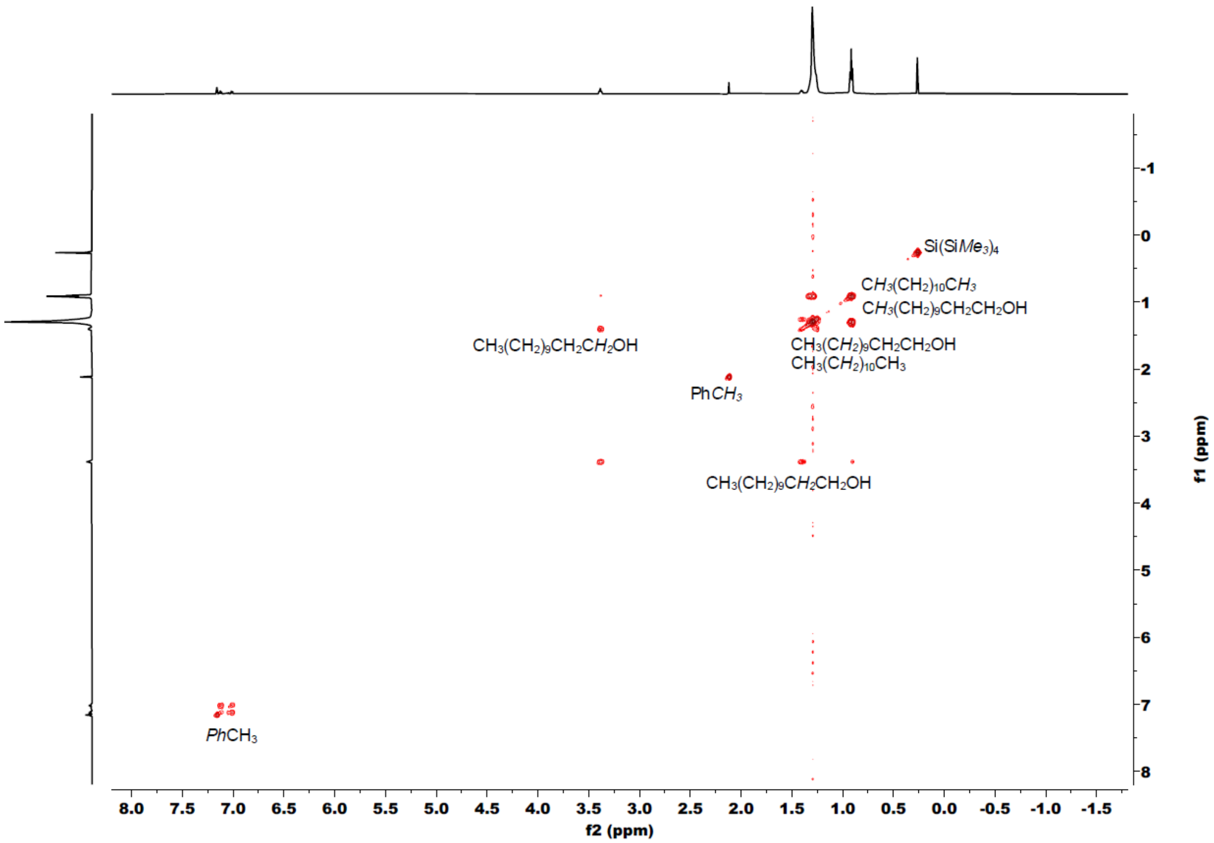

Figure S12. ^{1}H COSY NMR (600 MHz, benzene-d_6)) spectrum of the dodecan-1-ol and *n*-dodecane mixture obtained from the **1**-catalyzed (**1**: 30 mg, 0.019 mmol Zr) reaction of *n*-dodecane (1.3 mmol) and $AlEt_3$ (1.14 mmol; 60 equiv, with respect to Zr, 130.1 mg) for 12 h at 150 °C. $Si(SiMe_3)_4$ (25 mM, 1mL) was used as an internal standard.

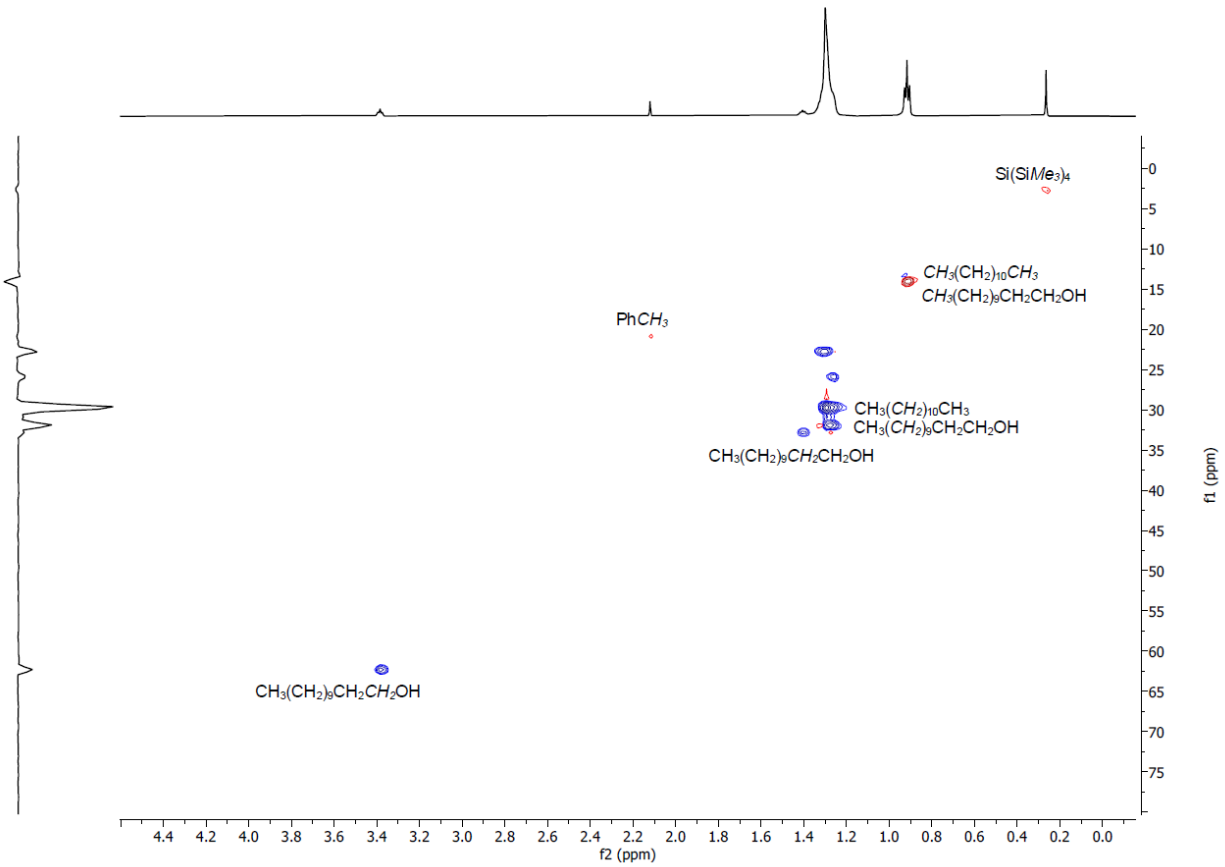

Figure S13. Multiplicity-edited ^{13}C-^{1}H HSQC NMR (150 MHz, benzene-d_6) spectrum of the dodecan-1-ol and *n*-dodecane mixture obtained from the **1**-catalyzed (**1**: 30 mg, 0.019 mmol Zr) reaction of *n*-dodecane (1.3 mmol) and $AlEt_3$ (1.14 mmol; 60 equiv, with respect to Zr, 130.1 mg) for 12 h at 150 °C. $Si(SiMe_3)_4$ (25 mM, 1mL) was used as an internal standard.

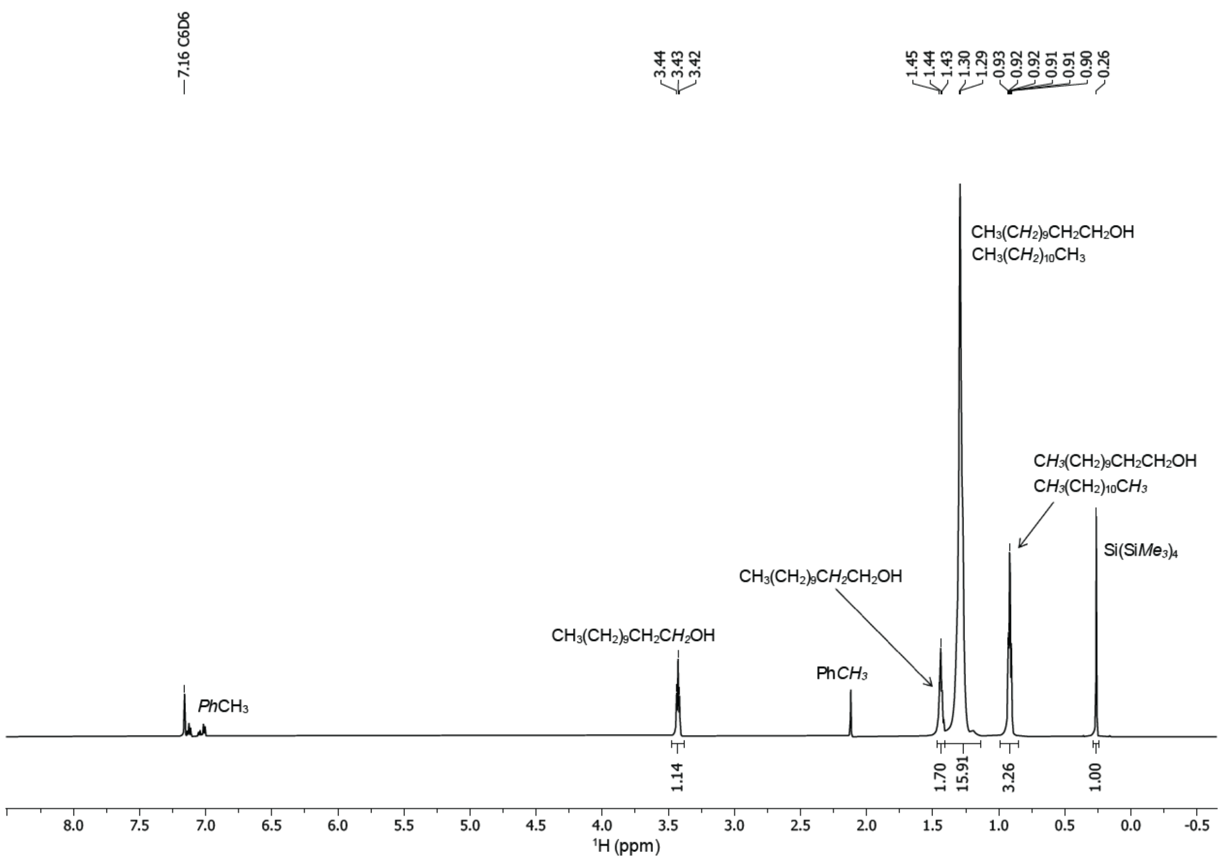

Figure S14. ^1H NMR (600 MHz, benzene-d_6) spectrum of dodecan-1-ol and *n*-dodecane mixture obtained from the **2**-catalyzed (**2**: 32 mg, 0.019 mmol Zr) reaction of *n*-dodecane (1.3 mmol) and $AlEt_3$ (4.0 mmol; 200 equiv with respect to Zr) for 15 h at 150 °C. $Si(SiMe_3)_4$ (25 mM, 1 mL) was used as an internal standard.

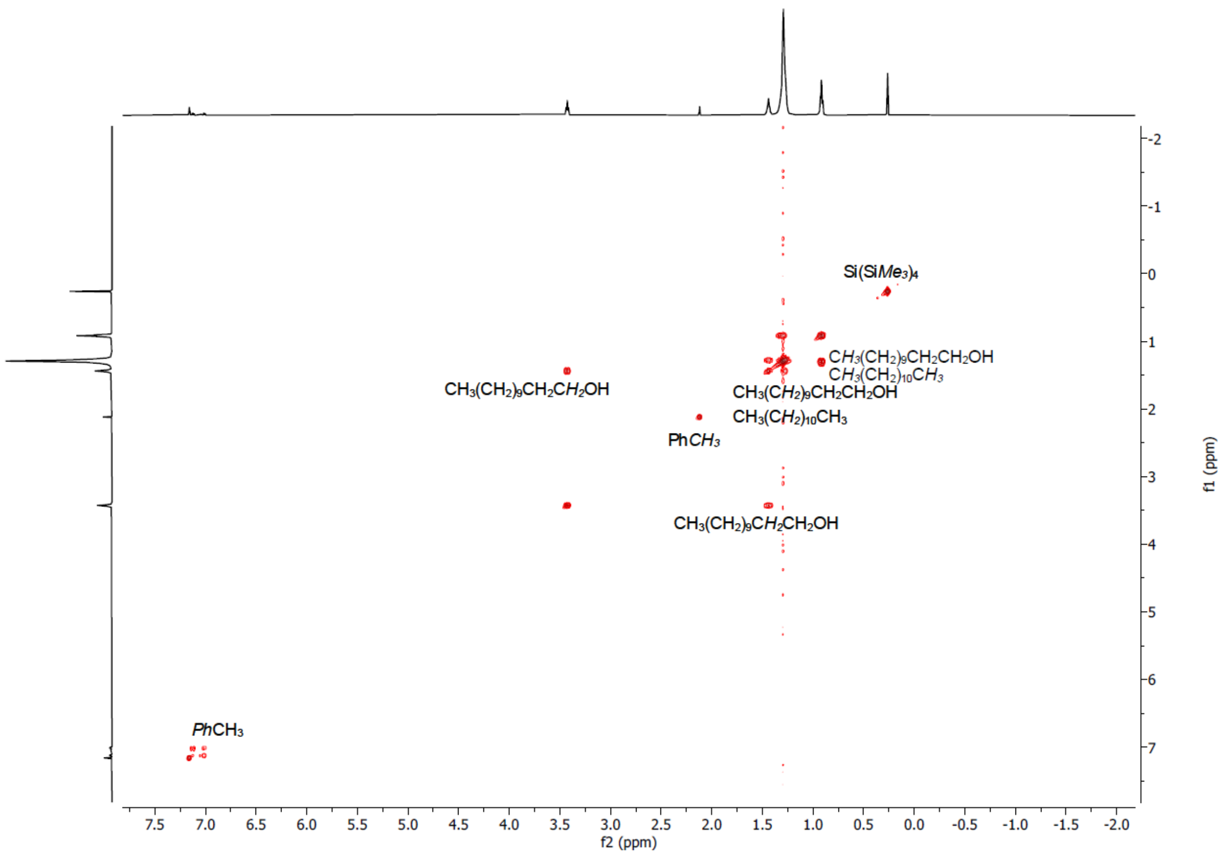

Figure S15. ^{1}H COSY NMR (600 MHz, benzene-d_6) spectrum of dodecan-1-ol and *n*-dodecane mixture obtained from the **2**-catalyzed (**2**: 32 mg, 0.019 mmol Zr) reaction of *n*-dodecane (1.3 mmol) and $AlEt_3$ (4.0 mmol; 200 equiv with respect to Zr) for 15 h at 150 °C. $Si(SiMe_3)_4$ (25 mM, 1 mL) was used as an internal standard.

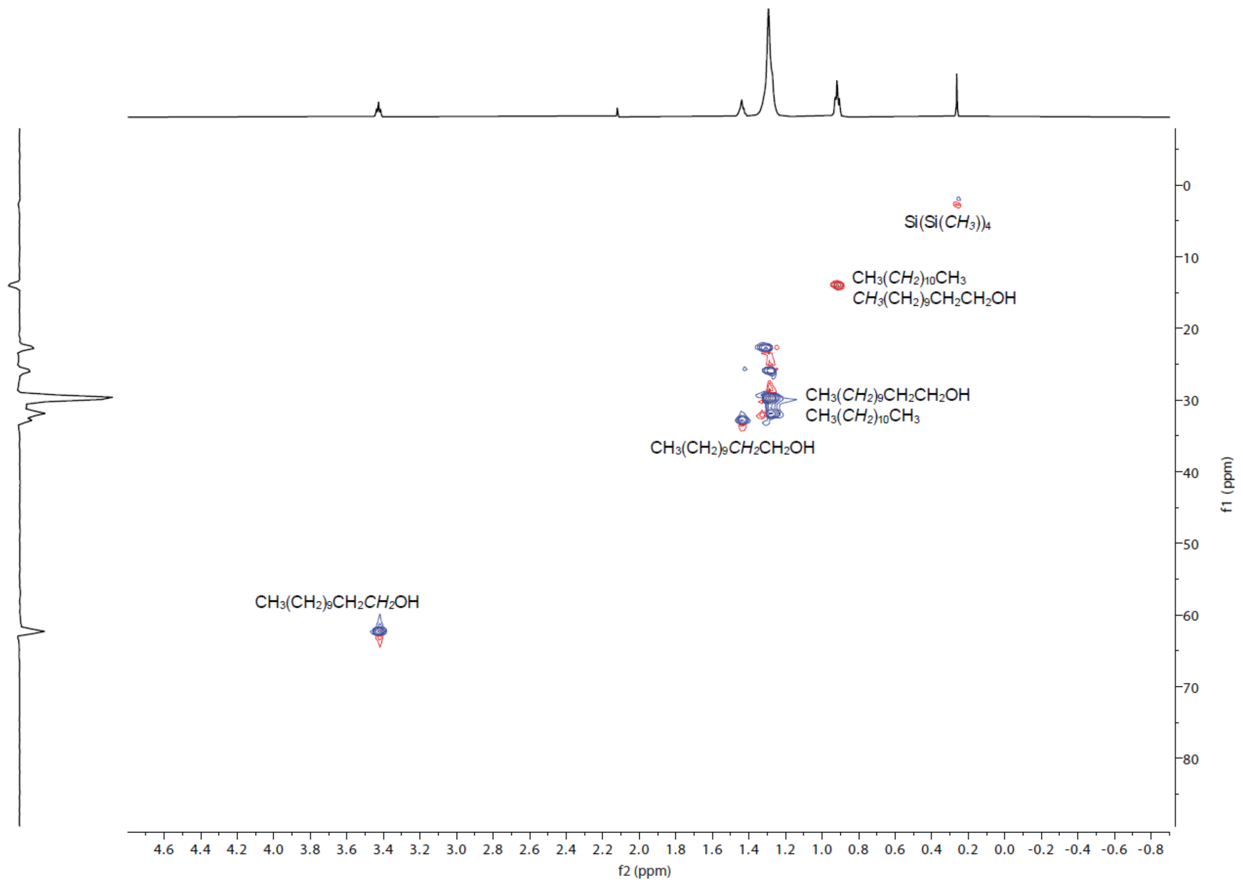

Figure S16. Multiplicity-edited ^{13}C-^{1}H HSQC NMR (150 MHz, benzene-d_6) spectrum of dodecan-1-ol and *n*-dodecane mixture obtained from the **2**-catalyzed (**2**: 32 mg, 0.019 mmol Zr) reaction of *n*-dodecane (1.3 mmol) and $AlEt_3$ (4.0 mmol; 200 equiv with respect to Zr) for 15 h at 150 °C. $Si(SiMe_3)_4$ (25 mM, 1 mL) was used as an internal standard.

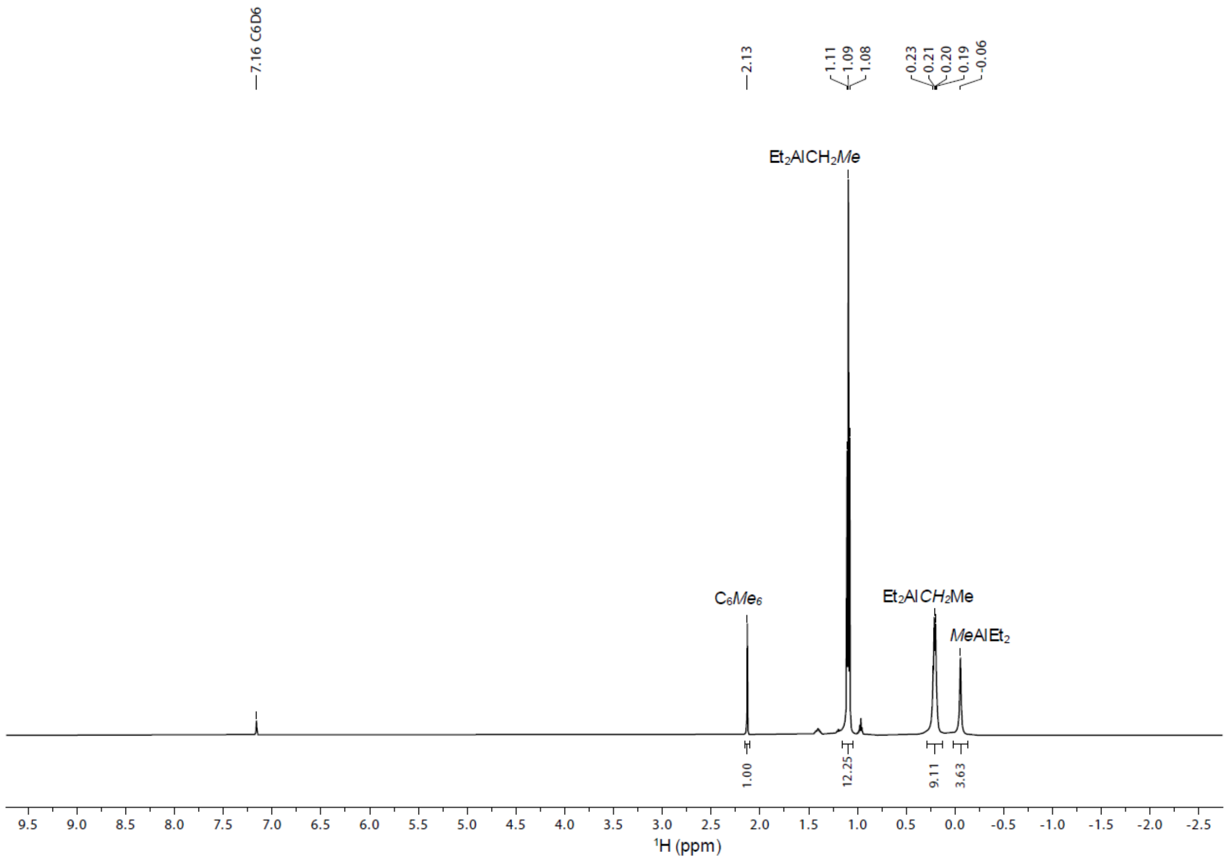

Figure S17. ^{1}H NMR (600 MHz, benzene-d_6) spectrum of the $AlEt_2Me$ and $AlEt_3$ mixture obtained from the **2**-catalyzed (**2**:32 mg, 0.019 mmol Zr) reaction of methane (725 psi) and $AlEt_3$ (1.14 mmol; 60 equiv with respect to Zr, 130 mg) for 15 h at 150 °C in presence of C_6Me_6 (5.8 mmol, 0.036 mg) as an internal standard. The signal at -0.06 ppm is the Et_2Al**Me** protons.

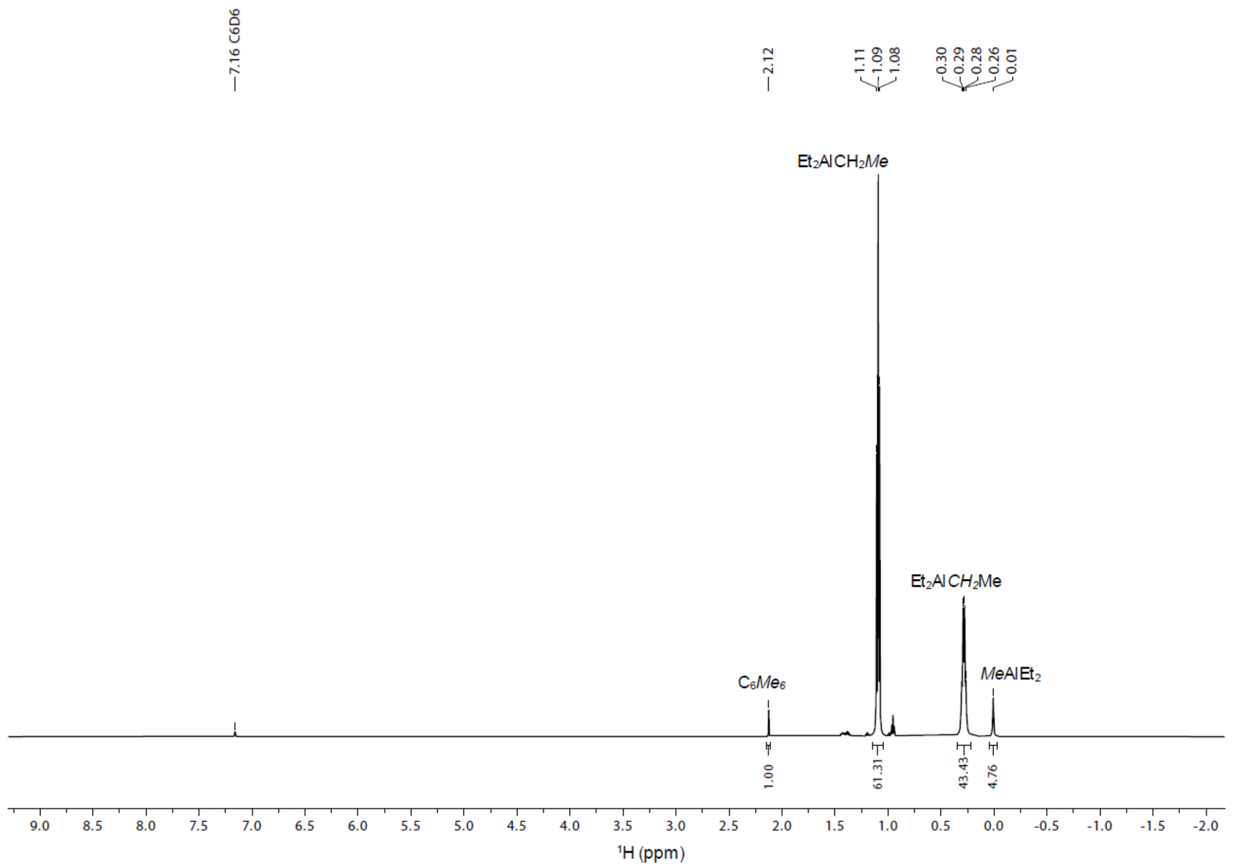

Figure S18. ^{1}H NMR (600 MHz, benzene-d_6) spectrum of the $AlEt_2Me$ and $AlEt_3$ mixture obtained from the **2**-catalyzed (**2**: 32 mg, 0.019 mmol Zr) reaction of methane (725 psi) and $AlEt_3$ (5.7 mmol; 300 equiv with respect to Zr, 650.7 mg) for 15 h at 150 °C in presence of C_6Me_6 (7.6 mg, 0.047 mmol) as an internal standard. The peak at 0.01 ppm is the Et_2Al**Me** protons.

GC trace of representative catalytic reactions

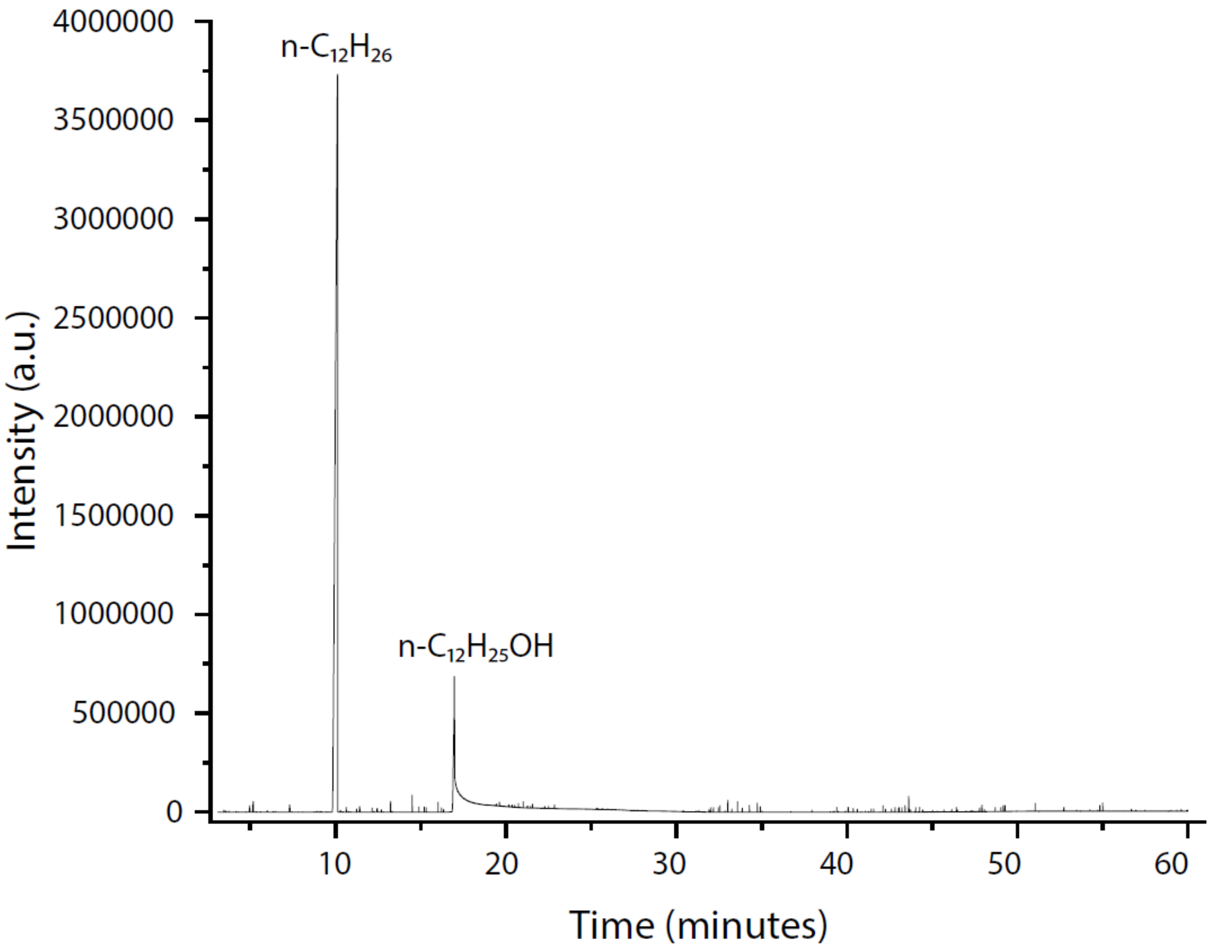

Figure S19. GC-MS of the reaction mixture of the catalysis of *n*-dodecane by **1** with $AlEt_3$ (60 equiv, with respect to Zr) at 150 °C.

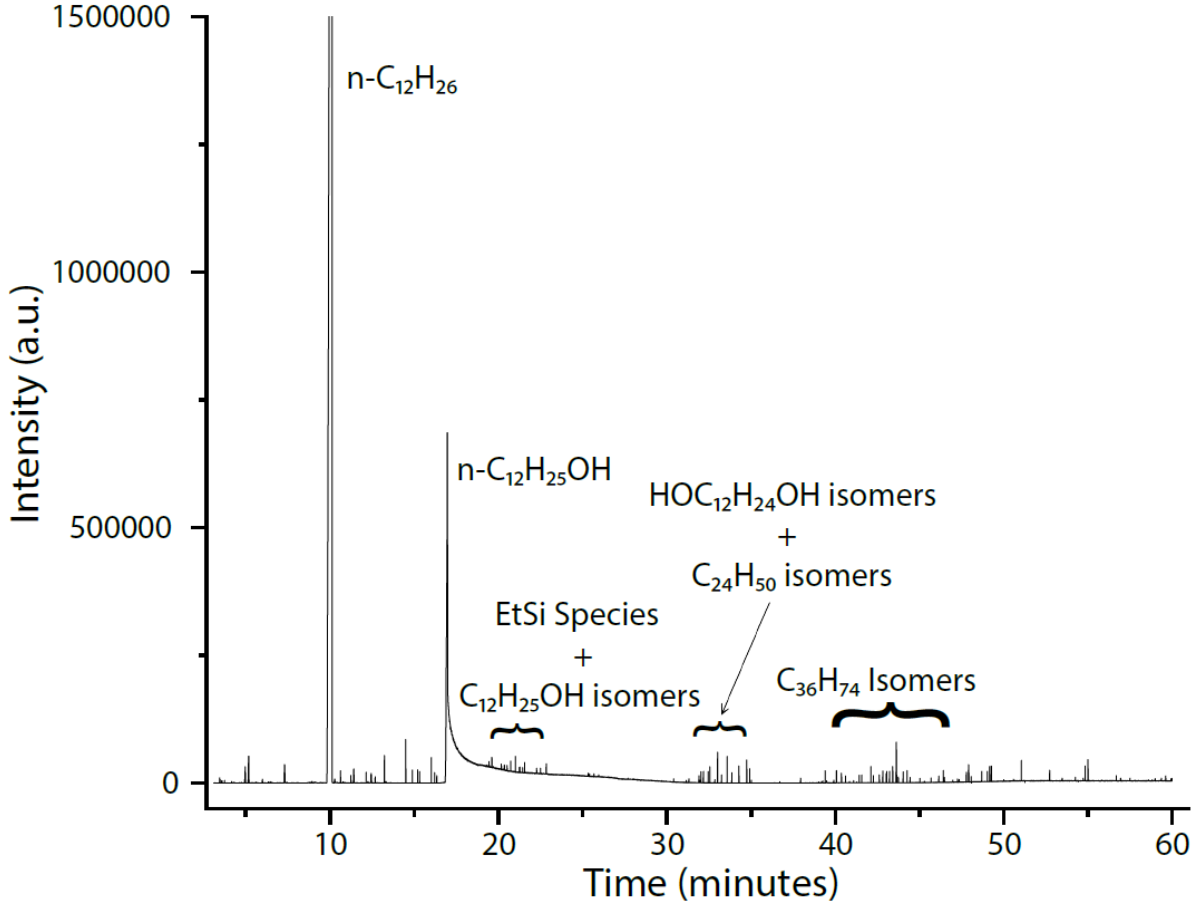

Figure S20. GC-MS (Figure S19 magnified to show the minor species) of the reaction mixture of the catalysis of *n*-dodecane by **1** with $AlEt_3$ (60 equiv, with respect to Zr) at 150 °C.

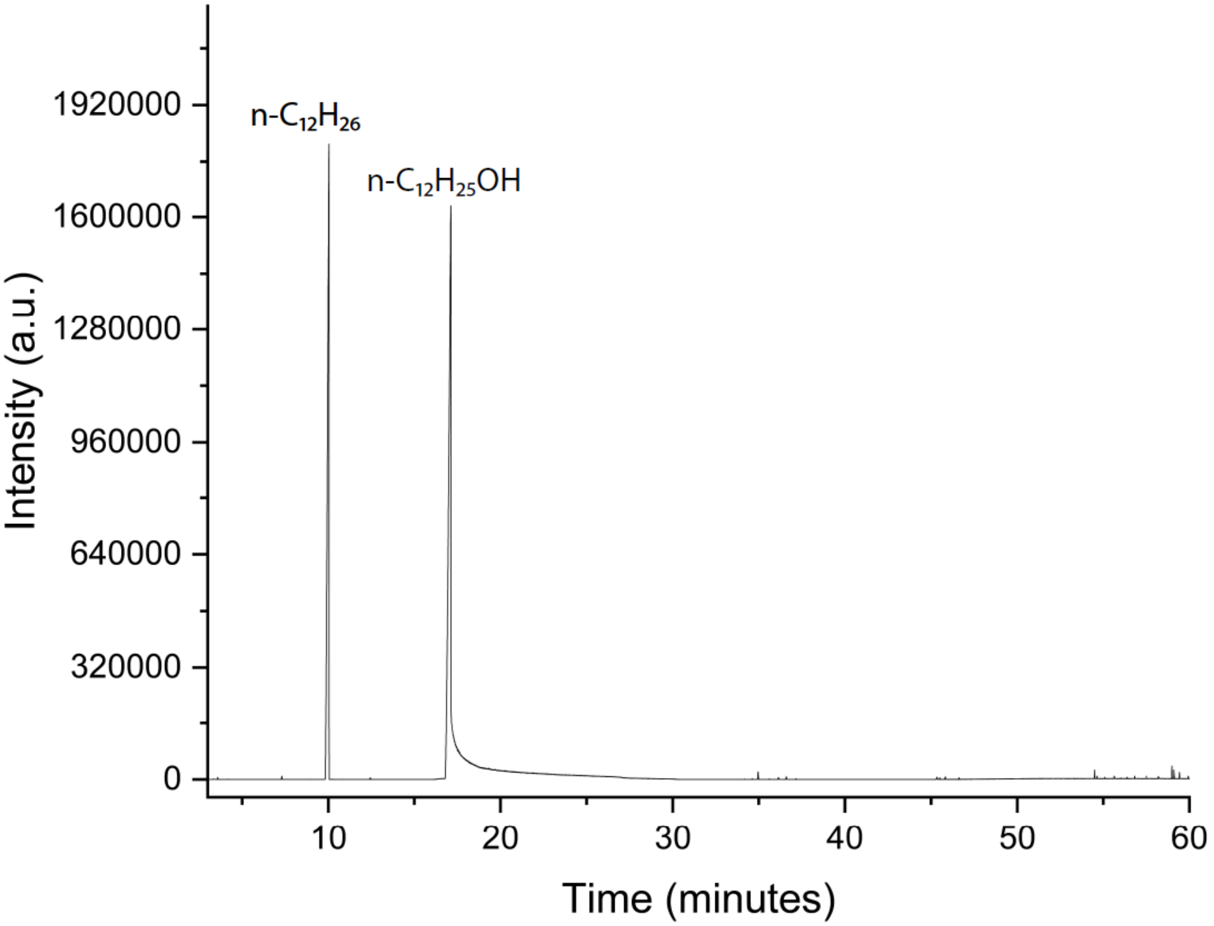

Figure S21. Representative GC-MS of the reaction mixture of the catalysis of *n*-dodecane by **2** with $AlEt_3$ (200 equiv, with respect to Zr) at 150 °C.

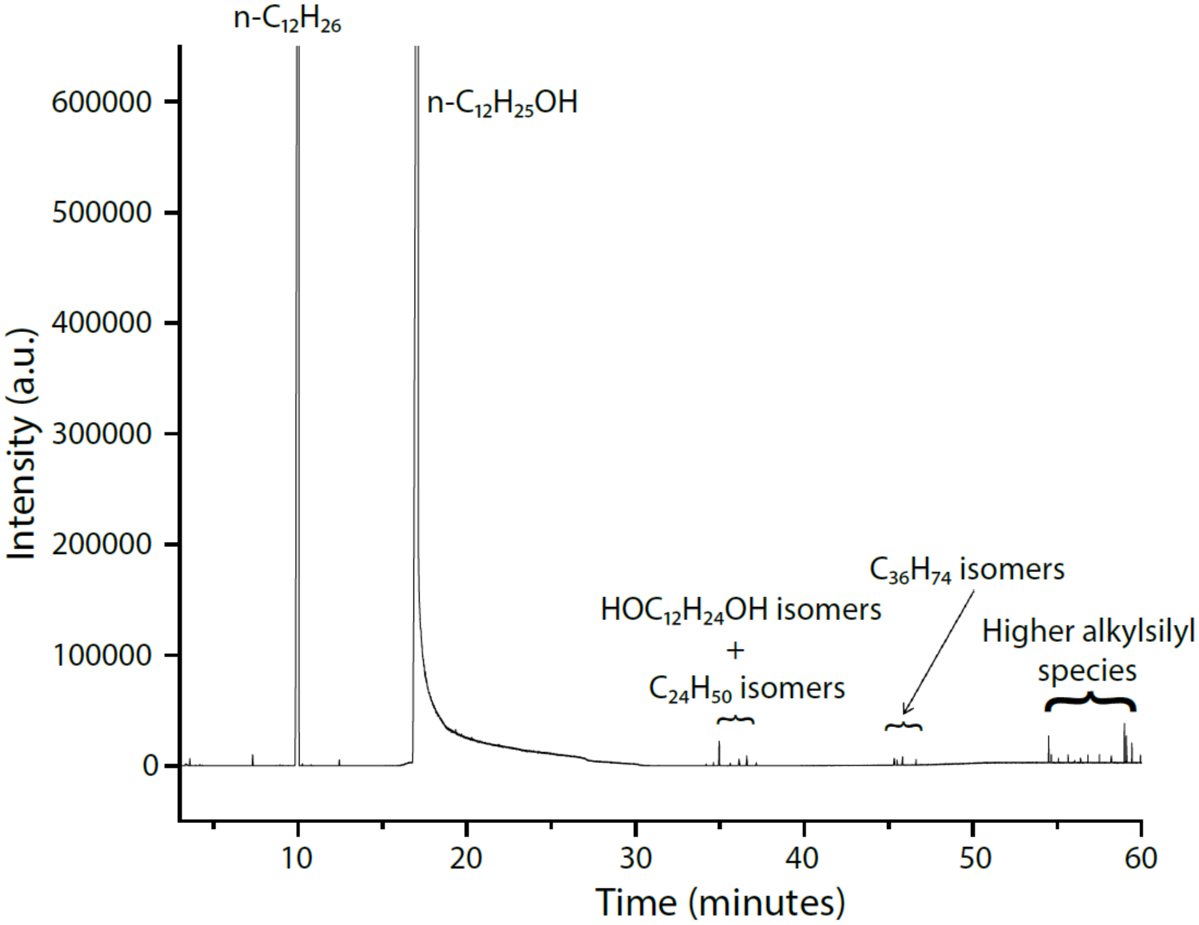

Figure S22. Representative GC-MS (Figure S21 magnified to show the minor species) of the reaction mixture of the catalysis of *n*-dodecane by **2** with $AlEt_3$ (200 equiv, with respect to Zr) at 150 °C.

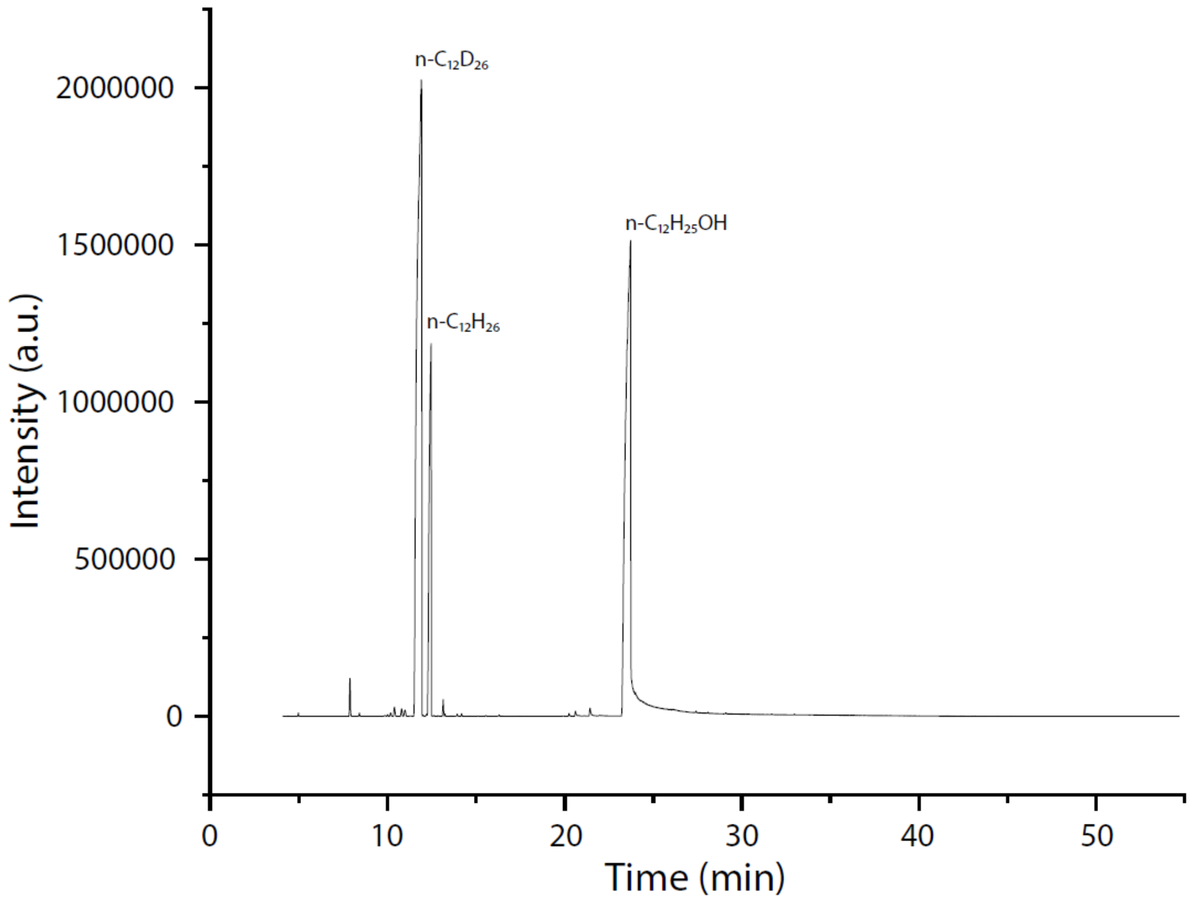

Figure S23. Representative GC-MS of the reaction mixture of the catalysis of *n*-dodecane (0.65 mmol) and n-dodecane-d_{26} (0.65 mmol) by **2** (32 mg, 0.019 mmol Zr) with $AlEt_3$ (120 equiv, with respect to Zr, 2.28 mmol, 260.3 mg) at 150 °C.

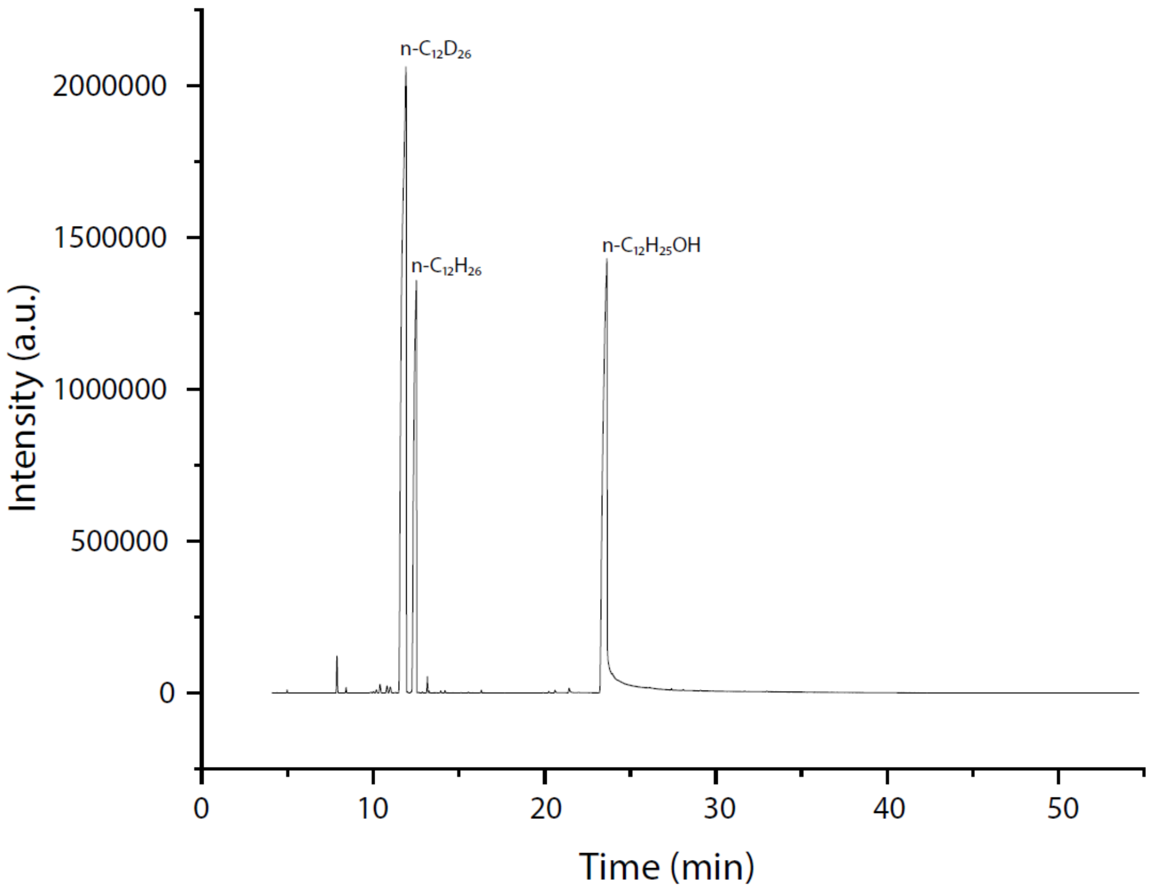

Figure S24. Representative GC-MS of the reaction mixture of the catalysis of *n*-dodecane (1.3 mmol) and n-dodecane-d_{26} (1.3 mmol) by **2** (32 mg, 0.019 mmol Zr) with $AlEt_3$ (120 equiv, with respect to Zr, 2.28 mmol, 260.3 mg) at 150 °C.

References

[1] J. T. Scanlon, D. E. Willis, "Calculation of Flame Ionization Detector Relative Response Factors Using the Effective Carbon Number Concept" *J. Chromatogr. Sci.* **1985**, *23*, 333-340.

[2] A. Zagdoun, A. J. Rossini, D. Gajan, A. Bourdolle, O. Ouari, M. Rosay, W. E. Maas, P. Tordo, M. Lelli, L. Emsley, A. Lesage, C. Copéret, "Non-aqueous solvents for DNP surface enhanced NMR spectroscopy" *Chem. Commun.* **2012**, *48*, 654-656.

[3] T. Kobayashi, F. A. Perras, U. Chaudhary, I. I. Slowing, W. Huang, A. D. Sadow, M. Pruski, "Improved Strategies for DNP-Enhanced 2D H-1-X Heteronuclear Correlation Spectroscopy of Surfaces" *Solid State Nucl. Magn. Reson.* **2017**, *87*, 38-44.

[4] A. Zagdoun, G. Casano, O. Ouari, M. Schwarzwälder, A. J. Rossini, F. Aussenac, M. Yulikov, G. Jeschke, C. Copéret, A. Lesage, P. Tordo, L. Emsley, "Large Molecular Weight

Nitroxide Biradicals Providing Efficient Dynamic Nuclear Polarization at Temperatures up to 200 K" *J. Am. Chem. Soc.* **2013**, *135*, 12790-12797.
[5] U. Kanbur, A. L. Paterson, J. Rodriguez, A. L. Kocen, R. Yappert, R. A. Hackler, Y.-Y. Wang, B. Peters, M. Delferro, A. M. LaPointe, G. W. Coates, F. A. Perras, A. D. Sadow, "Zirconium-Catalyzed C–H Alumination of Polyolefins, Paraffins, and Methane" *J. Am. Chem. Soc.* **2023**, *145*, 2901-2910.
